# Temporal Ordering in Endocytic Clathrin-Coated Vesicle Formation via AP2 Phosphorylation

**DOI:** 10.1016/j.devcel.2019.07.017

**Published:** 2019-08-19

**Authors:** Antoni G. Wrobel, Zuzana Kadlecova, Jan Kamenicky, Ji-Chun Yang, Torsten Herrmann, Bernard T. Kelly, Airlie J. McCoy, Philip R. Evans, Stephen Martin, Stefan Müller, Filip Sroubek, David Neuhaus, Stefan Honing, David J. Owen

**Affiliations:** 1 CIMR, WT/MRC Building, Hills Road, Cambridge CB2 0QQ, UK; 2 Czech Academy of Sciences, Institute of Information Theory and Automation, Pod Vodarenskou vezi 4, 182 08 Prague 8, Czech Republic; 3 MRC Laboratory of Molecular Biology, Cambridge Biomedical Campus, Francis Crick Avenue, Cambridge CB2 0QH, UK; 4 University of Grenoble Alpes, CNRS, CEA, IBS, 38000 Grenoble, France; 5 The Francis Crick Institute, 1 Midland Road, London NW1 1ST, UK; 6 Center for Molecular Medicine (CMMC), University of Cologne, Robert-Koch-Straße 21, 50931 Cologne, Germany; 7 Institute for Biochemistry I, Medical Faulty, University of Cologne, Joseph-Stelzmann-Straße 52, 50931 Cologne, Germany

**Keywords:** clathrin-mediated endocytosis, regulation by phosphorylation, AP2 endocytic adaptor, NECAP, SNX9, AAK1, Numb-associated kinases (NAK), NMR, crystallography, TIRF

## Abstract

Clathrin-mediated endocytosis (CME) is key to maintaining the transmembrane protein composition of cells’ limiting membranes. During mammalian CME, a reversible phosphorylation event occurs on Thr156 of the μ2 subunit of the main endocytic clathrin adaptor, AP2. We show that this phosphorylation event starts during clathrin-coated pit (CCP) initiation and increases throughout CCP lifetime. μ2Thr156 phosphorylation favors a new, cargo-bound conformation of AP2 and simultaneously creates a binding platform for the endocytic NECAP proteins but without significantly altering AP2’s cargo affinity *in vitro*. We describe the structural bases of both. NECAP arrival at CCPs parallels that of clathrin and increases with μ2Thr156 phosphorylation. In turn, NECAP recruits drivers of late stages of CCP formation, including SNX9, via a site distinct from where NECAP binds AP2. Disruption of the different modules of this phosphorylation-based temporal regulatory system results in CCP maturation being delayed and/or stalled, hence impairing global rates of CME.

## Highlights

• AP2 μ2T156 phosphorylation starts early at CCP inception and increases as CCP grows
• Phosphorylation favors an AP2 conformational change and also triggers NECAP binding
• NECAP PHear simultaneously binds P-AP2 and membrane-remodeling proteins on opposite faces
• Disrupting the P-AP2:NECAP:membrane-remodeling protein network reduces CME rates

## Graphical Abstract

**Figure:**
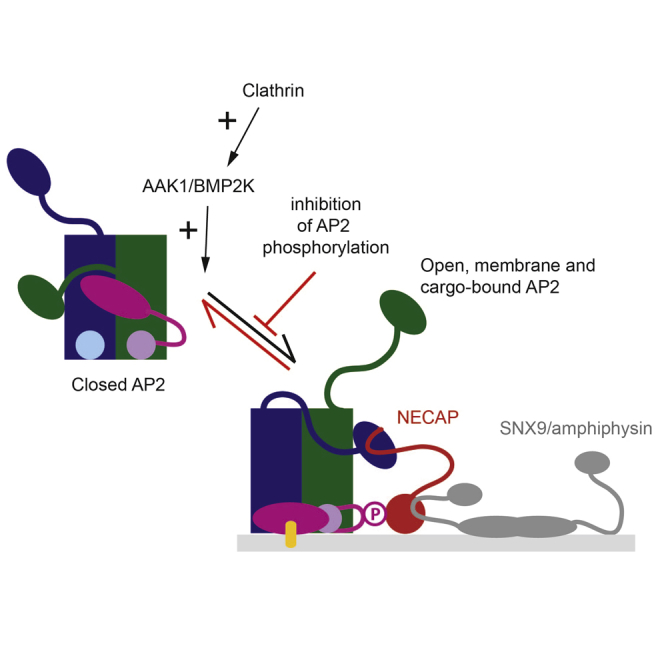

## Teaser

Wrobel et al. show that phosphorylation of the mammalian endocytic AP2 adaptor causes a conformational change that allows it to efficiently bind the protein NECAP. This, in turn, recruits membrane-remodeling proteins into clathrin-coated pits, which drive their formation toward final scission from the parent membrane.

## Introduction

The levels of a vast array of transmembrane proteins of widely differing functions must be tightly but dynamically regulated. This is achieved by controlling the rate of each protein’s internalization, which occurs mainly by clathrin-mediated endocytosis (CME) and balancing it with its rates of delivery to the surface after biosynthesis, transport down the endocytic system, and recycling back to the cell surface. CME is mediated by clathrin-coated vesicles (CCVs) that matured from clathrin-coated pits (CCPs). The most abundant proteins in endocytic CCVs are clathrin itself, which forms the outer polyhedral scaffold, and the clathrin adaptors AP2 and clathrin assembly lymphoid myeloid leukemia protein (CALM), which couple the clathrin layer to the inner membrane. Lower levels of other clathrin adaptors and CME accessory proteins are also found in the central layer (Borner et al., 2012, Kirchhausen et al., 2014). Other proteins that can be localized to CCPs are found in vanishingly small quantities in intact CCVs (Borner et al., 2012).

Early biochemical studies, supported by more recent live-cell imaging and cryo-electron tomography studies (Heuser, 1980, Kukulski et al., 2012), have resulted in a model for coat formation of single, isolated, CCVs, (reviewed in Lampe et al., 2016, Mettlen et al., 2018) in which formation proceeds in an ordered, stepwise manner: initiation at a site enriched in PtdIns4,5P_2_ by AP2 aided by Fcho1/2 and Eps15/R that can be stabilized by clathrin recruitment, followed by expansion of a clathrin-coated structure through recruitment of more cargo-bound clathrin adaptors and clathrin, and finally coat and membrane deformation. Similar CCVs can also be formed from the periphery of large, flat pre-existing clathrin “plaques” in an analogous manner (Lampe et al., 2016). In either case, deformation continues to form a “necked” structure that undergoes scission and then finally uncoating (reviewed in Antonny et al., 2016, Mettlen et al., 2018).

Reversible protein phosphorylation is a common mechanism for controlling cellular processes (Nishi et al., 2011). The multiplicity of kinases and phosphatases, whose activity can be regulated in a wide number of ways including by phosphorylation itself, allows this mode of regulation to be finely tuned with exquisite temporal and spatial precision and also to be arranged in amplification cascades and feedback loops. Protein phosphorylation often exerts regulation through driving conformational changes, creating binding determinants for positively charged moieties, and/or repelling poly-anionic species such as the negatively charged phospholipid-rich membranes, as demonstrated by the neuronal dephosphins (Cousin and Robinson, 2001).

The 300 kDa heterotetrameric AP2 complex (α, β2, μ2, and σ2 subunits) (Figure S1A) is essential for CCV formation. Binding to PtdIns4,5P_2_ causes recruitment of AP2 to the plasma membrane, which induces a conformational change from its closed cytosolic form (Collins et al., 2002), resulting in activation of two key functions: binding of the two most common transmembrane protein endocytic cargo-sorting signals (YxxΦ and [ED]xxxLL) and binding to clathrin (Jackson et al., 2010, Kelly et al., 2008, Owen and Evans, 1998). Thus, clathrin polymerization and coat formation are coupled to cargo capture (Kadlecova et al., 2017, Kelly et al., 2014).

AP2 is subject to multiple phosphorylation events (Conner and Schmid, 2002, Olusanya et al., 2001, Pauloin et al., 1982, Pauloin and Thurieau, 1993, Wilde and Brodsky, 1996). The best characterized and most prominent site of phosphorylation in AP2 is T156 in the linker between the two domains of μ2 (Höning et al., 2005, Olusanya et al., 2001, Pauloin and Thurieau, 1993, Smythe and Ayscough, 2003). The effects of mutation of μ2T156 *in vivo* combined with biophysical studies from our laboratories suggested that phosphorylation was required for cargo capture via a phosphorylation-induced conformational change that facilitates cargo and membrane binding. However, this hypothesis is based largely on *in vitro* studies, and it conflicts with aspects of temporal ordering in CCV formation. For instance, phosphorylation of μ2T156 by a Numb-associated kinase (NAK) family member, either α-adaptin associated kinase (AAK1) or potentially BIKE (aka BMP2K) (Borner et al., 2012, Sorrell et al., 2016), is thought to be activated by clathrin binding (Conner and Schmid, 2002, Jackson et al., 2003, Olusanya et al., 2001, Ricotta et al., 2002, Sorrell et al., 2016). Consequently, μ2 phosphorylation should only occur after some clathrin polymerization on AP2 has already happened, and this in turn will only have occurred when AP2 has already bound membrane and cargo and so undergone its conformational change (Jackson et al., 2010, Kelly et al., 2014).

To unravel the functional significance of μ2 phosphorylation, we applied an array of complementary approaches: quantitative immunofluorescence was used to localize phosphorylated AP2 to CCPs; X-ray crystallography to determine a new open conformation adopted by phosphorylated AP2 (termed P-AP2 from now on); surface plasmon resonance (SPR) to measure the affinity of AP2 and P-AP2 to endocytic signals; biochemical and mass-spectrometry-based analyses to identify and characterize the phosphorylation-dependent binding of a new μ2 interaction partner, NECAP; and structural NMR analysis to characterize this interaction at the molecular level. Finally, highly specific NAK kinase inhibitors were used in combination with high temporal resolution, multi-color, live-cell imaging to show that AP2 phosphorylation is required for efficient NECAP recruitment to CCPs and the maturation of productive CCVs.

## Results

### Molecular Perturbations of AP2 μ2 Phosphorylation in RPE Cells

**Figure 1:**
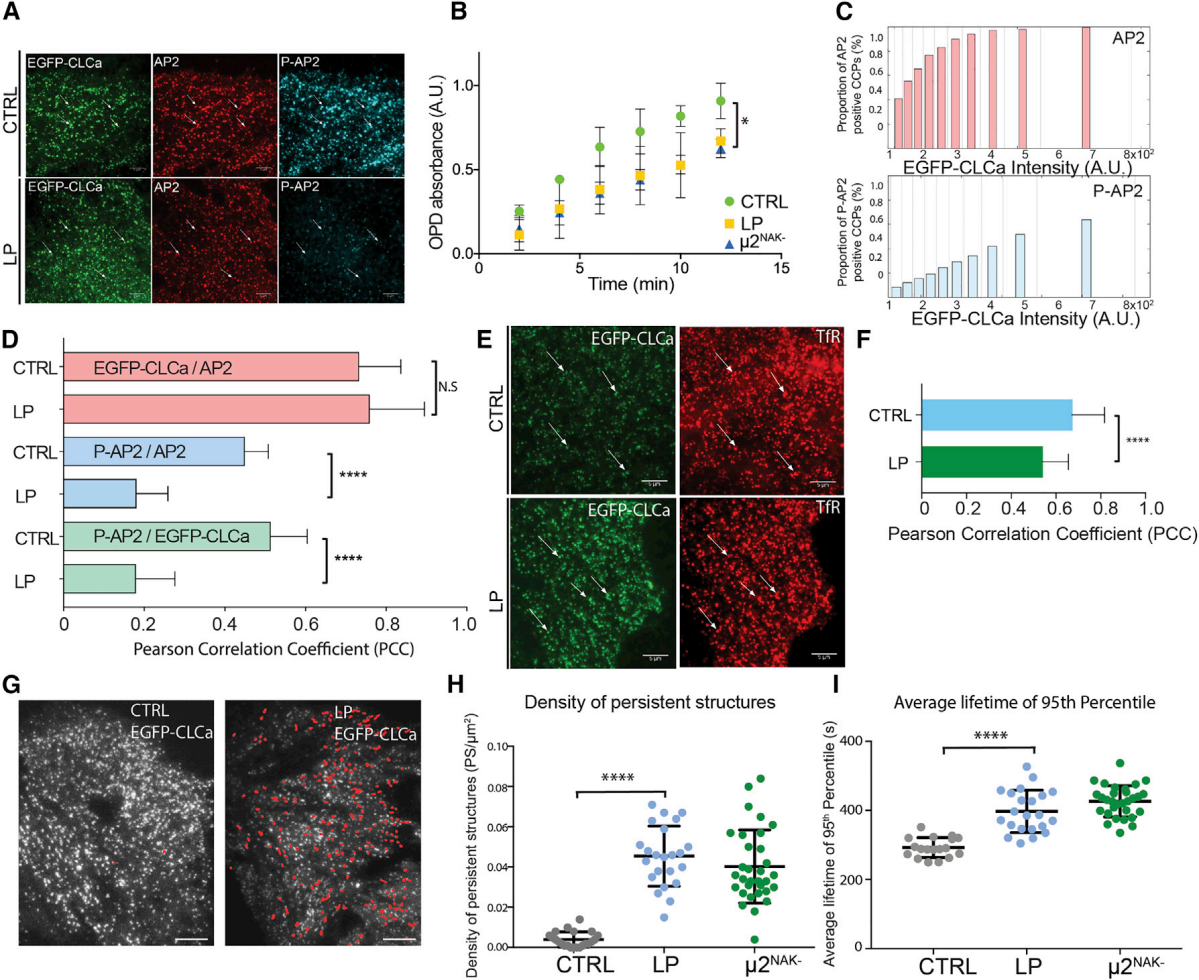
μ2T156 Phosphorylation Increases as CCPs Mature and Is Important for Efficient CME (A) Representative images of control RPE cells and cells treated with LP. White arrows indicate the presence of a fluorescent signal in the green channel (EGFP-CLCa) and the same position in the AP2 (red) and P-AP2 channel (cyan) to compare presence or absence of the corresponding signal at the same position. Scale bar, 5 μm. (B) Time course of TfR internalization at 37°C. Data represent mean SD, n = 3. Two-tailed unpaired t tests were used to assess statistical significance. ^∗^p ≤ 0.05. (C) Bar graphs represent the quantification of the membrane distribution of AP2 (red) or P-AP2 (blue) in CCPs. The bar height displays the proportion of CCPs (detected in the EGFP-CLCa channel) that contain significant AP2 or P-AP2 signal in the same position plotted as a function of EGFP-CLCa signal (x axis). Data represent mean values, n = 30. (D) Comparison of PCCs to assess the relationship of the paired intensity values of CCP components EGFP-CLCa, AP2, and P-AP2. Data represent mean SD, n = 30. Two-tailed unpaired t tests were used to assess statistical significance. ^∗∗∗∗^p ≤ 0.0001; ns, p > 0.05. (E) Representative images of control cells and cells treated with LP, showing the distribution of EGFP-CLCa signal and TfR detected with anti-TfR mAb. White arrows indicate the presence of the signal in the green channel (EGFP-CLCa) and the same position in the TfR channel. Scale bar, 5 μm. (F) PCCs comparing the strength of linear relationship of the paired intensity values of EGFP-CLCa and TfR. Bars represent mean SD, n = 27. Two-tailed unpaired t tests were used to assess statistical significance. ^∗∗∗∗^p ≤ 0.0001. (G) Representative TIRFM images of control cells and cells treated with LP. Red highlights indicate clathrin-coated structures categorized as persistent. Scale bar, 5 μm. (H and I) Scatterplots with the values of density of the persistent structures (H) and the average lifetime of the 95th percentile (I) for control cells, cells treated with LP, and μ2^NAK−^ RPE cells. Lines represent mean and SD for each group (n = 20). Two-tailed unpaired t tests were used to assess statistical significance. ^∗∗∗∗^p ≤ 0.0001.

The specific localization and even the partitioning between cytosol and membrane fractions of P-AP2 in unperturbed cells has long been controversial, although it was recently shown that AP2, rendered cytosolic by mutation of its PtdIns4,5P_2_ binding site, showed markedly reduced levels of μ2 phosphorylation (Kadlecova et al., 2017). Here, we use an antibody that only recognizes μ2T156 when it is phosphorylated (Figure S1B and manufacturer’s information). Immunofluorescence microscopy of retinal pigment epithelium cells (RPE) cells shows that P-AP2 is present in the majority (∼70%) of AP2-positive CCPs (Figure 1A).

Small-molecule inhibitors allow for acute inhibition of kinase activity and hence minimize compensatory pathways that may obscure the actual function of phosphorylation events. LP-935509 (subsequently LP) is a recently evolved potent (low nM IC50) inhibitor of the μ2-phosphorylating NAK family members in human AAK1 and BIKE, which binds in their structurally similar ATP-binding pockets (Kostich et al., 2016, Sorrell et al., 2016). We used LP and also a previously tested structurally different inhibitor (INHIB2 CAS 1093222-27-5) (Bamborough et al., 2008) to analyze the dependence of CCP formation and rates of CME on AP2 phosphorylation in RPE cells. Treatment with either inhibitor for 3 h effectively decreased AP2 phosphorylation by ∼80% (Figure S1B). As LP is well characterized and since it has very few off-target effects (Kostich et al., 2016), LP alone was used in all subsequent experiments. We also established a mutant RPE cell line, expressing an AP2 μ2-subunit in which the consensus substrate recognition sequence was mutated at its key −2 (Q to S) and +1 (G to A) positions (termed AP2μ2^NAK−^) (Smythe and Ayscough, 2003). Use of AP2μ2^NAK−^ allowed us to completely ablate AP2 phosphorylation while maintaining physicochemical and biochemical properties of T156 (Figures S1C and S1D).

Next, we assessed the dependency of CME rates on AP2 phosphorylation by following transferrin receptor (TfR) internalization: its rate decreased by approximately 30% in both inhibitor-treated cells and also in μ2^NAK−^ cells (Figures 1B and S1D). μ2^NAK−^ cells incubated with the inhibitor LP showed no further decrease of TfR internalization efficiency as compared with untreated μ2^NAK−^ cells (Figure S1F), indicating that the phenotype elicited by NAK kinase inhibitors can be attributed to the loss of μ2T156 phosphorylation and not to an off-target effect of inhibitors.

### The Quantity of P-AP2 in CCPs Positively Correlates with CCP Growth

Using quantitative immunofluorescence microscopy, we first analyzed global distribution of total AP2 and P-AP2 in CCPs and calculated the proportion of CCPs that contain AP2 and P-AP2. We plotted these values as a function of the respective EGFP-CLCa intensities in the detections (Figure 1C). In control cells, the proportion of detections positive for P-AP2 increases linearly with the quantity of EGFP-CLCa. CCPs that contain P-AP2 are fewer than the CCPs that contain AP2 for any cohort with a given EGFP-CLCa intensity. Inhibition of NAK kinases with LP reduces the number of CCPs that contain P-AP2 by at least ∼3-fold in all clathrin-intensity cohorts (Figure S1G). Assuming that EGFP-CLCa intensity at CCPs increases during the growth phase of a CCP (Loerke et al., 2011), our results suggest that AP2 phosphorylation occurs at the plasma membrane and its quantity increases with CCP age.

The correlation analysis of paired fluorescence intensity values between P-AP2, AP2, and EGFP-CLCa reveals that the quantity of P-AP2 and EGFP-CLCa and the quantity of P-AP2 and total AP2 are positively correlated in CCPs. In comparison, the correlation between the quantity of AP2 and EGFP-CLCa is even higher (Figure 1D). These data indicate that the quantity of P-AP2 in a CCP is influenced by a factor other than the quantity of AP2 and EGFP-CLCa. We speculate that it reflects the regulated activity and quantity of NAK in CCPs. The correlation between the total quantity of AP2 and EGFP-CLCa was independent of the quantity of μ2T156 phosphorylation. These data suggest that μ2T156 phosphorylation is unlikely to directly affect the interactions of AP2 with either the plasma membrane or clathrin. Further, these data indicate another role for AP2 phosphorylation in CCP formation. As expected, the correlation between P-AP2 and AP2 and between P-AP2 and EGFP-CLCa dropped by ∼3-fold upon inhibition of μ2T156 phosphorylation with LP (Figure 1D).

Previous studies used several different experimental systems to test the role of AP2 phosphorylation in cargo recruitment to CCPs and yielded conflicting results (reviewed in Smythe and Ayscough, 2003). Figures 1E and 1F show that the proportion of CCPs that are TfR positive is not affected by the phosphorylation status of μ2T156, while the accumulation of TfR in CCPs decreases by only 20% if the NAK activity is ablated (Figure 1F).

### Phosphorylation of AP2 Is Necessary for Efficient Maturation of CCPs

We used quantitative live-cell total internal reflection fluorescence microscopy (TIRFM) to investigate which stage of CCP assembly fails in the absence of phosphorylation and compared CCP assembly rates in inhibitor-treated cells and control cells. Inhibition of μ2T156 phosphorylation resulted in a ∼10-fold increase in the density of diffraction-limited clathrin-coated structures that failed to pinch off during the 10-min video acquisition time (persistent structures) (Figures 1G and 1H). We also observed that the population of CCPs that underwent scission displayed extended lifetimes (Figures 1I and S1H).

In sum, these data indicate that μ2T156 phosphorylation affects CCP maturation and the rate of CME but not likely due to alterations in the efficiency of cargo sequestration.

### Structures of μ2T156-P-AP2 Core

Recombinant T156-phosphorylated AP2 core (Pcore) with >95% stoichiometrically phosphorylated T156 was produced as in Höning et al., 2005. Initially, we determined the structure of Pcore in the closed inactive form at an ∼2.8-Å resolution in complex with the PtdIns4,5P_2_ analog IP6 by molecular replacement using the closed structure (PDB 2VGL) as a search model (Table S1). The structure did not differ from that of the unphosphorylated AP2 (core) (root-mean-square deviation [RMSD] of 0.56 Å over 1,717 residues). No electron density for the phosphorylated μ2 linker was visible, suggesting that it does not bind back onto the rest of the core and that it is statically and/or dynamically disordered.

Despite extensive efforts, we were unable to crystallize Pcore in the presence of the TGN38 YxxΦ cargo peptide DYQRLN in any conditions, including those that led to the determination of the initial open structure where the myc tag of an adjacent AP2 takes the place of an [ED]xxxLL cargo motif (Jackson et al., 2010). However, in the presence of both DYQRLN and RM(pS)QIKRLLSE, crystals were obtained that diffracted to an apparent resolution of 3.4 Å with an anisotropic deltaB of 1.6. The structure was solved by two-stage molecular replacement with Phaser using first the “bowl” (α and β2 solenoids, Nμ2, and σ2) of the open form (PDB 2XA7) and subsequently Cμ2 from the same structure (McCoy, 2007, Winn et al., 2011), and as with all previous AP2 structures, some regions are considerably better ordered than others.

**Figure 2:**
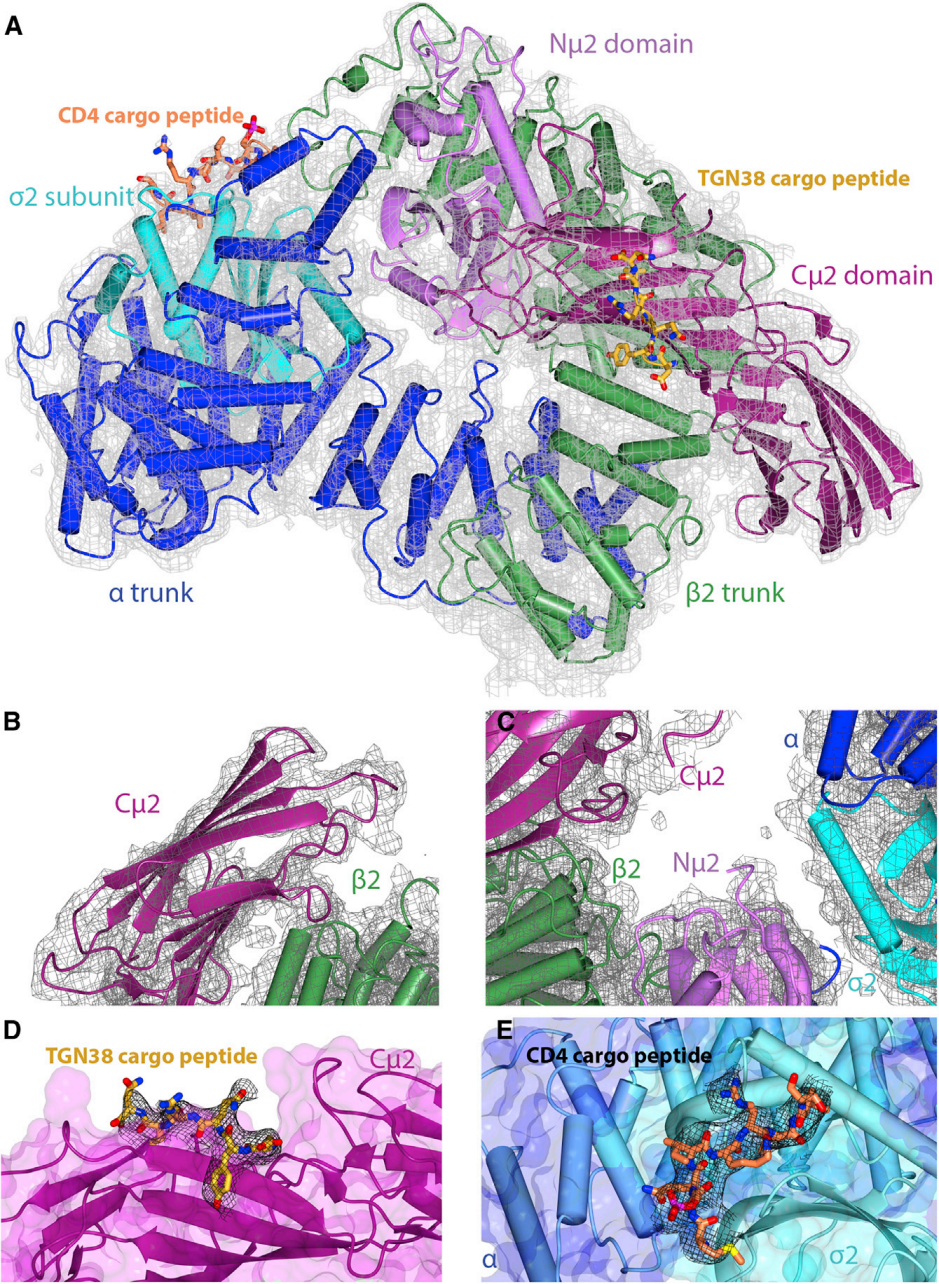
A New Open+ Conformation for Cargo-Bound AP2 in Both μ2t156 Phosphorylated and Unphosphorylated Forms (A) Structure of T156-phosphorylated AP2 core (Pcore) in open+ conformation with YxxΦ cargo in gold and dileucine cargo in orange. The refined 2mFo-DFc electron density map (gray) is contoured at 1 sigma. (B–E) Enlargements of functionally important regions with subunits and refined 2mFo-DFc electron density colored as above: (B) shows unambiguous Cμ2 positioning, (C) the missing portion of the phosphorylated μ2 linker, (D) YxxΦ cargo peptide (2mFo-DFc map clipped to 3Å around peptide, contoured at 1 sigma), and (E) dileucine cargo peptide (2mFo-DFc map clipped to 3Å around peptide, contoured at 0.5 sigma).

**Figure 3:**
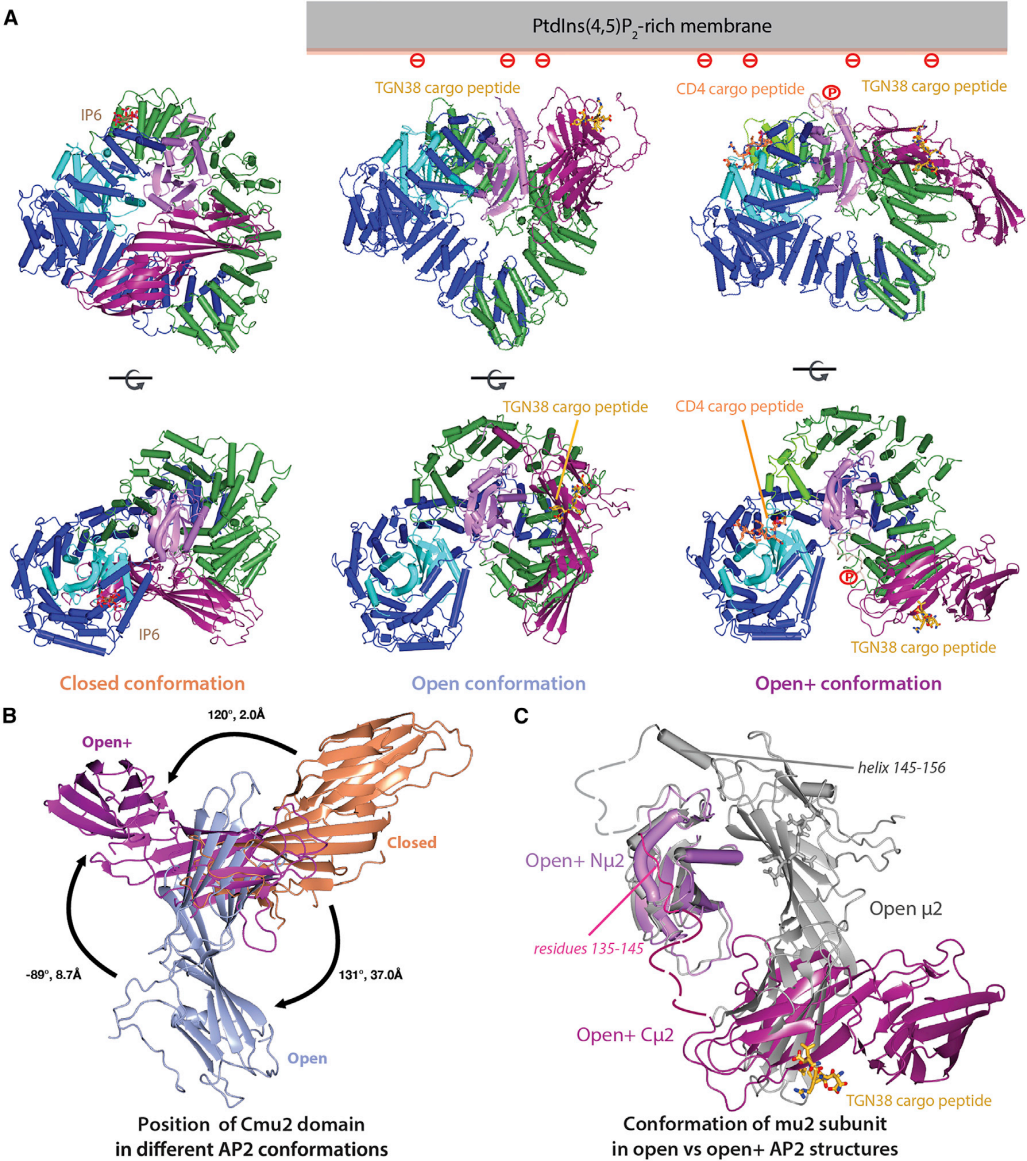
The Open+ Conformation Is Related to the Open Conformation by Movement of μ2 and Is Membrane- or Cargo-Binding Competent (A) Views “perpendicular to” (top row) and “through” (bottom row) the membrane of Pcore in “closed” or “locked” cytosolic conformation (left column) and membrane attached open (center) and open+ (right column) conformations. (B) Relative positions of Cμ2 in closed (orange), open (blue), and open+ (purple) conformations of Pcore aligned by the superposition of their α subunits. (C) Superposition of entire μ2 subunits in open (gray) and open+ conformations, superimposed on the basis of Nμ2 to highlight the change in orientation of the two subdomains relative to each other viewed “through the membrane.” Modeled fragments of linkers are shown as dashed.

The structure is clearly in a new active and open conformation that we term open+ (Table S1; Figures 2A–2C and S2A): the bowl superimposes well (RMSD of 1.3Å over 1,353 residues) with that of the open form, and the hollow of the bowl is not occupied by Cμ2, as it is in the closed conformation (Figures S2A–S2C). Both cargo peptides are clearly bound (Figures 2D–2E and S2B–S2D), although the electron density would suggest the [ED]xxxL[LI] binding site is not fully occupied. The main difference with the open conformer (PDB 2XA7) is that Cμ2 is in a different orientation, rotated by ∼90° and translated by ∼9Å with respect to N-μ2 such that it packs against residues 300–400 of β2, burying a solvent accessible surface area of 640Å^2^ (Figures 2A, 3B, 3C, S2A–S2D, and S3A–S3F). There are further differences between the two conformations; for instance, in the new conformation, the first four helices of β2 are poorly ordered although present (see Supplemental Information). The loss of electron density for these helices can be explained by the breaking of the interface between the α/σ2 and β2/Nμ2 heterodimers since in the closed conformation, a large part of this interface comes from F6 and Y7 of β2 binding into the dileucine-motif-binding site on σ2, which is now displaced by genuine dileucine cargo. This new open+ conformation indicates that Cμ2 is able to explore the membrane in its local vicinity to “hunt” for cargo whilst remaining physically tethered to the membrane-binding bowl through the μ2 linker.

The μ2 linker in the open form (2XA7) is mostly ordered with residues 142–157 adopting a helix. T156 is buried and so inaccessible to a kinase and hence should not be phosphorylatable. Further, a phosphate at this position would also likely cause steric clashes with β2 and Nμ2, which would disfavor an open conformation for Pcore and explain our inability to crystallize Pcore in the open conformation. In the new open+ form, there is no traceable density for the residues 134–158 including the phosphorylated threonine (Figure 2C) despite using various approaches (see STAR Methods). N.b., there had been no change in the percentage of phosphorylated μ2T156 as determined by analysis of Pcore post-data collection (data not shown): these data suggest that in open+, the m2 linker is likely unstructured, mobile, and accessible to NAK kinases and T156-phosphate when present and does not bind back onto AP2 to drive the conformational change as we and others had suggested (Höning et al., 2005, Olusanya et al., 2001).

Crystals with similar morphology and space group dimension to those of open+ Pcore were also obtained of core, but none diffracted beyond an ∼6-Å resolution. Molecular replacement clearly showed that the core in this crystal form was in the open+ conformation (Figures 3A, S3C, and S3D; Table S1): this was not pursued further. These data suggest, however, that the open+ conformation is accessible to both core and Pcore but is likely promoted by μ2T156 phosphorylation. Thus, it is likely that in the cell, unphosphorylated AP2 exists in equilibrium between open and open+ (and possibly other) forms (Figure 3D) with only the open+ form compatible with μ2T156 phosphorylation.

### μ2T156 Phosphorylation Does Not Have a Major Effect on AP2’s Affinity for Membrane-Embedded Cargo

The open+ structure is compatible with membrane-embedded cargo binding, although it would need to use some different lysine residues on Cμ2 for PtdIns4,5P_2_ binding (Figure S2D). In light of this and the improvements in SPR, we revisited our liposome-based SPR studies (Höning et al., 2005). The association phases were too complicated to be modeled; however, the dissociation phases could be modeled using a double exponential, corresponding to fast and slow phases (Table S2; Figure S3G). Binding to PtdIns4,5P_2_-only membranes is unaffected by the core phosphorylation state: the fast and slow phases likely correspond to closed forms associating via one of their two orthogonal PtdIns4,5P_2_-binding sites. The presence of cargo increases the apparent strength of AP2 or P-AP2 binding to all PtdIns4,5P_2_-containing liposomes, presumably by triggering the AP2 or P-AP2 to adopt open or open+ conformations. The fast and slow phases now seen result from AP2 or P-AP2 bound to PtdIns4,5P_2_ only and to PtdIns4,5P_2_ + cargo, respectively, with the interactions being 5- to 15-fold (fast) and 30- to 100-fold (slow) stronger than the fast and slow phases seen when cargo is not present. However, there is only a modest (∼3-fold) increases in YxxΦ-containing liposome-binding affinity on core phosphorylation, likely resulting from phosphorylation stabilizing the open+-cargo-binding competent core conformation. There are no obvious differences upon AP2 phosphorylation in binding the dileucine cargo. In support of this, using fluorescence anisotropy, we show that μ2T156 phosphorylation causes no obvious change in the affinity of AP2 for YxxΦ cargo peptides, when AP2 is stimulated by the addition of polyanionic heparin to mimic the presence of a PtdIns4,5P_2_-enriched membrane (Figure S3H) (Jackson et al., 2010).

When considered with the effects of treatment with NAK inhibitors described above, these data suggest that although phosphorylation of μ2T156 is functionally important during endocytosis, it is not through significantly altering the affinity of AP2 for the membrane and its embedded cargo as we and others had previously suggested. The question thus arises, “what is the major role of μ2T156 phosphorylation in CME?” One obvious possibility is that μ2 phosphorylation could modulate or even be a prerequisite for binding of other proteins.

### μ2T156 P-AP2 Cores Bind to NECAP1 and NECAP2

**Figure 4:**
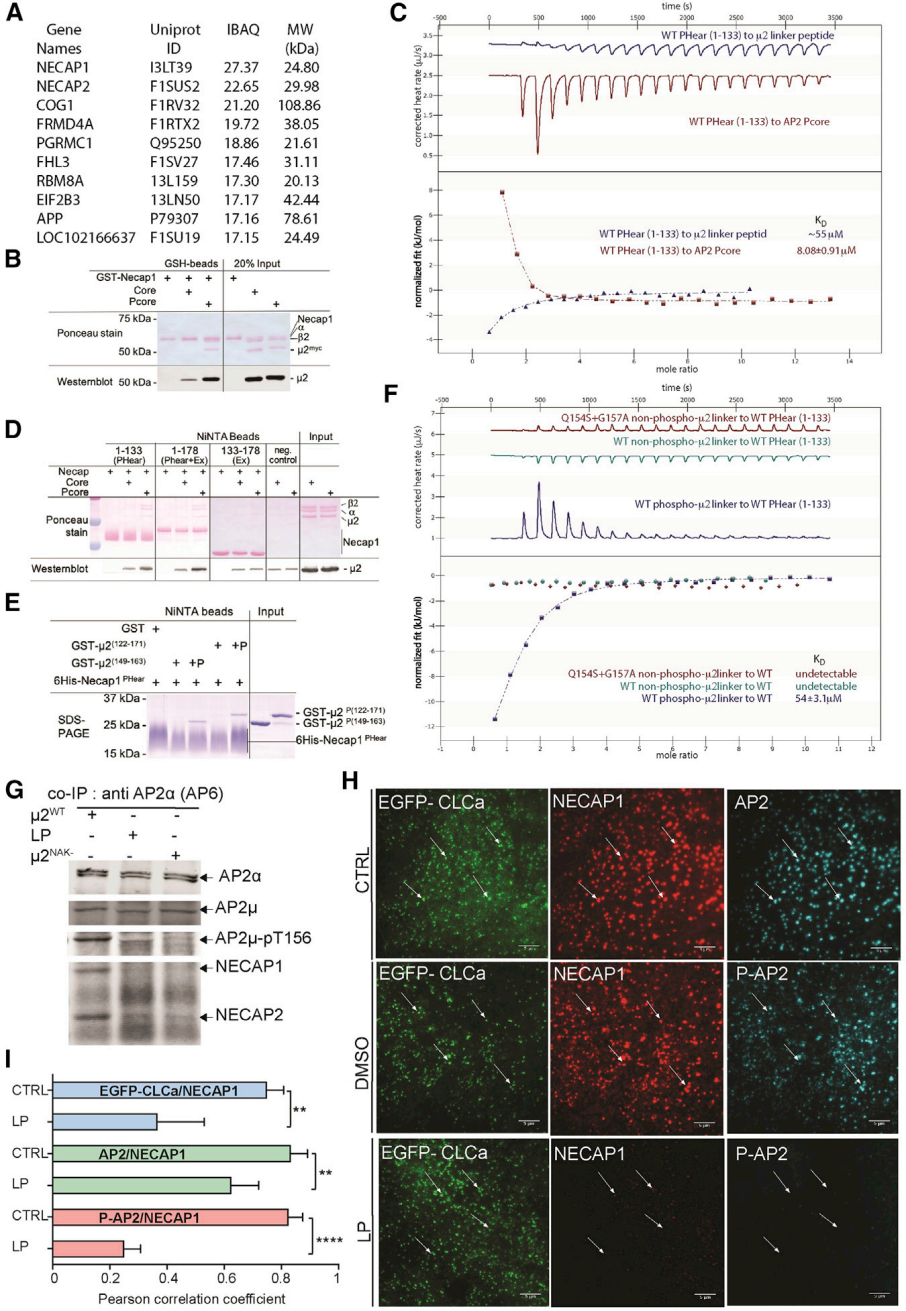
NECAP Binds to AP2 Core in a μ2T156-Phosphorylation-Dependent Manner (A) Table showing the 10 proteins form pig brain cytosol as scored by their iBAQ values that are most enriched in their binding to GST-Pcore over GST-core. The top two “hits” are NECAP1 and NECAP2. (B) Myc-tagged Pcore preferentially binds to full-length recombinant NECAP in pull-downs. SDS- PAGE gels were blotted and stained with Ponceau red (upper) or developed with anti-myc antibody (lower). (C) Pcore binds more tightly to NECAP1 PHear than the phosphorylated μ2 linker. Representative ITC traces (top) and fitted curves with K_D_s of binding (bottom) of Pcore in red and to μ2-linker (residues 149–163)-derived peptide (cell) in blue to WT NECAP1 PHear (syringe). (D) SDS-PAGE gels of “pull-downs” using His_6_NECAP1 constructs of various length immobilized on NiNTA beads were blotted and stained with Ponceau red (upper panels) and developed with anti-myc antibody (lower panel), showing that PHear and PHear-Ex but not Ex bind to Pcore. (E) SDS-PAGE gels of “pull-downs” using His_6_NECAP1 PHear immobilized on NiNTA beads shows that NECAP PHear binds GSTμ2 linkers 122–171 and 149–163 only when T156 is phosphorylated by co-expression of linkers with the AAK1 catalytic subunit. (F) NECAP1 PHear only binds μ2 linker peptides when the peptides are phosphorylated: K_D_ of 55 μM. Example ITC traces (top) and fitted curves with K_D_s of binding (bottom) of WT NECAP1 PHear (cell) to μ2-linker- (residues 149–163) derived peptides (syringe): Q154S + G157A (kinase ablation) mutant, red; WT unphosphorylated, cyan; and T156-phosphorylated, dark blue. (G) Comparison of the quantity of endogenous NECAP that co-immunoprecipitated with AP2 from lysates obtained from control cells (first lane) and cells treated with LP and μ2^NAK−^ RPE cells (middle and last lane) by western blotting with anti NECAP polyclonal antibody. AP2 binds NECAPs only when AP2 is μ2T156 phosphorylated. (H) Representative immunofluorescence images showing co-localization of EGFP-CLCa, NECAP1, and AP2 (top row) and EGFP-CLCa, NECAP1, and P-AP2 when treated with DMSO (middle row) or LP (bottom row). White arrows indicate the presence of a fluorescent signal in the EGFP-CLCa channel and the same position in the NECAP (red channel) and AP2 or P-AP2 channel (cyan channel). Scale bar, 5 μm. (I) Comparison of PCCs to assess the relationship of the paired intensity values of CCP components EGFP-CLCa, NECAP, AP2, and P-AP2 from immunofluorescence images. Data represent the mean SD, n = 30. Two-tailed Student’s t tests were used to assess statistical significance. ^∗∗^p ≤ 0.01, ^∗∗∗∗^p ≤ 0.0001.

To search for potential Pcore binding partners, we carried out pull-down experiments from brain lysates using glutathione S-transferase (GST)-tagged core and Pcore coupled to sepharose beads and mass spectrometry for protein identification. Any protein with binding to GST and/or core was rigorously filtered from the data. In the remaining list, NECAP1 and NECAP2 were identified as the two top hits that bound Pcore only (Figure 4A; Table S3). These data were confirmed in subsequent pull-down experiments with pure recombinant proteins (Figures 4B, S4C, and S4D), which showed that NECAP1 preferentially binds Pcore over core. These data are in line with a recent elegant study in *C. elegans* (Beacham et al., 2018), published after our independent identification of NECAP as a direct binding partner of mammalian P-AP2 had been made.

### P-AP2 Binds NECAPs through Its Phosphorylated μ2 Linker and α-Appendage

NECAPs consist of an N-terminal, 133-residue PHear domain, and C-terminal 120–150 residues unstructured region that contains WxxF α-appendage binding motifs (Figures S4A and S4B) in mammalian but not in *C. elegans* NECAP orthologs. NECAPs are known to bind other endocytic proteins containing FxDxΦ-like motifs, such as amphiphysin or Bin1 and CALM with K_D_s in a range of hundreds of μM as determined in NMR titrations (Ritter et al., 2007), indirectly influence CCV size via CALM (Miller et al., 2015, Ritter et al., 2013), and a loss-of-function mutation in *necap1* is a heritable cause of early infantile epileptic encephalopathy (Alazami et al., 2014).

“Pull-downs” and isothermal titration microcalorimetry (ITC) (Figures 4C, 4D, and S4C–S4E) show that the PHear domain alone binds Pcore but not core in solution with a K_D_ of ∼8 μM. In these experiments, the Pcore should be in its closed non-cargo-binding form (this work and (Jackson et al., 2010)). Using μ2 constructs of various lengths, either unphosphorylated or phosphorylated by co-expressing them with the AAK1 kinase domain, we showed that the μ2 linker (residues 122–163) is sufficient for an interaction with NECAP PHear and that the interaction is direct and completely dependent on phosphorylation of μ2T156 (Figure 4E).

The phosphorylated μ2 linker is largely invisible, i.e., disordered and accessible in the open+ as well as in the closed Pcore structures and thus should be able to bind NECAP PHear domains in both conformations. ITC using a phosphorylated μ2 linker synthetic peptide of 15 residues (SQITSQV(pT)GQIGWRR) and NECAP1 PHear domain demonstrated that the interaction possessed a K_D_ ∼55 μM (Figures 4F and S4E). No binding could be detected for non-phosphorylated μ2^WT^ or μ2^NAK−^ linker peptides (Figure 4F), control threonine phosphorylated peptide IKIIDEK(pT)GVIEHE, or free phosphothreonine (Figure S4F), indicating that the binding of the isolated linker to PHear was largely mediated by the phosphothreonine but only occurred when it was presented in the correct context. The strength of binding of the isolated phosphorylated linker is somewhat weaker than that of the phosphorylated linker when it is part of Pcore, in agreement with the “pull-down” data (Figure S4D). This difference suggests the existence of another, albeit weak, determinant for PHear binding, on closed Pcore at least. Whether this additional site also exists on a cargo-binding competent form of Pcore, we cannot say.

A high μM K_D_ interaction between the AP2 α-appendage and a WxxF motif at the C terminus of mammalian NECAP-1 has been characterized (Praefcke et al., 2004; our unpublished data). To explore the contribution of both sites of interaction, the WxxF motif and PHear as part of full-length NECAP, we used a p-AP2 core that contains α-appendage separated from the α-trunk by a synthetic linker of the correct length, which circumvents its rapid proteolysis. The presence of both cognate binding surfaces on both partners caused avidity effects and result in a comparatively stronger interaction (Figure S4D) and hence a NECAP:AP2 complex could be isolated by gel filtration (data not shown). Although our attempts to crystallize such a complex sadly proved unsuccessful, these data indicate that the α-ear binding could provide a further determinant in addition to the phosphorylated μ2 linker to augment P-AP2’s ability to bind or recruit NECAP in mammalian cells but not in *C. elegans*.

### Efficient Recruitment of NECAPs to CCPs Is Dependent on Phosphorylation of AP2

To explore the dependency of the interaction between NECAPs and AP2 on its phosphorylation, we used native co-immunoprecipitation of endogenous proteins from RPE cell lysates under low-detergent conditions. In RPE cells, both NECAP1 and NECAP2 interact with P-AP2. We found that in all conditions the NECAP1:AP2 and NECAP2:AP2 interaction was nearly undetectable in the absence of μ2T156 phosphorylation, resulting from LP inhibitor treatment or using the μ2^NAK−^ mutant (Figure 4G).

To investigate if there was a correlation between the phosphorylation of AP2 and presence of NECAP1-mRuby2 (Figure S4G) in CCPs, we used quantitative analysis of immunofluorescence images (Figure 4H). We observed a strong positive correlation between the quantity of NECAP1-mRuby2 and EGFP-CLCa and total AP2 and P-AP2 in CCPs. Treatment with LP inhibitor significantly reduces the quantity correlation between each pair of CCP components, with the largest drop being between NECAP1-mRuby2 and P-AP2 (Figure 4I). The histograms in Figure S4H show that the proportion of CCPs positive for NECAP1-mRuby2 increases with the amount of EGFP-CLCa. The same is true for the NECAP1-mRuby2 and total AP2 and NECAP1-mRuby2 and P-AP2. The probability of finding NECAP1-mRuby2 increases most rapidly with the quantity of P-AP2. LP inhibitor treatment reduced the probabilities in all three cases (Figure S4H).

### NECAP Is Recruited to CCPs Early and Regulates CCP Maturation

**Figure 5:**
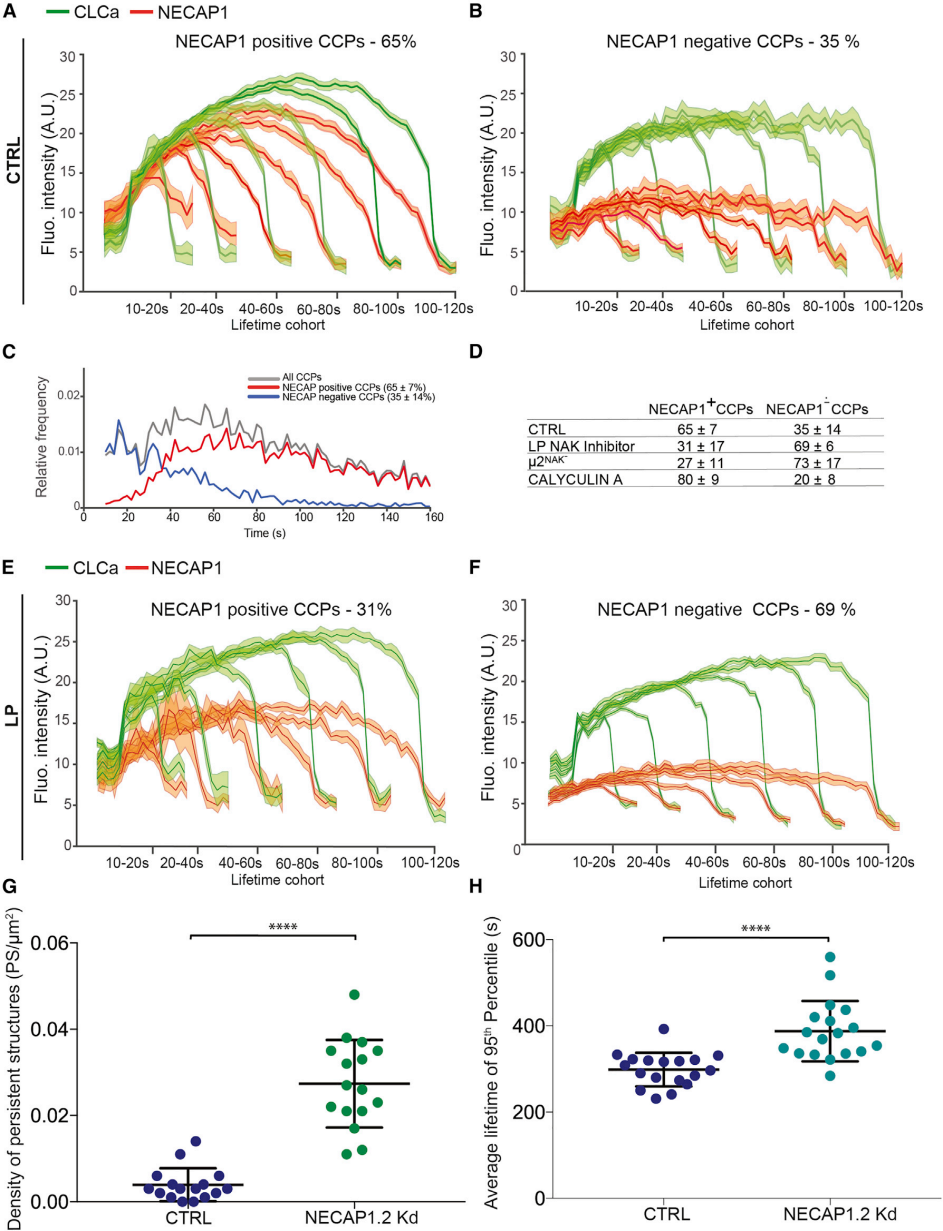
A μ2T156 Phosphorylation Drives NECAP Recruitment to CCPs (A and B) CCPs detected in control RPE cells expressing EGFP-CLCa and NECAP1-mRuby2 were grouped into cohorts with a specific range of lifetimes, and corresponding intensity traces of EGFP-CLCa and NECAP1-mRuby2 were plotted as a function of the time. CCPs were further subcategorized into NECAP-positive CCPs (A) and NECAP-negative CCPs (B). Averaged CCP intensity traces for EGFP-CLCa (green) and NECAP1 (red) from control cells are shown as mean ± SE (shaded areas) per lifetime cohort. (C) Lifetime distributions of all CCPs (gray) in control RPE cells expressing EGFP-CLCa and NECAP1-mRuby2. CCPs were further subcategorized as NECAP1-positive (red) or NECAP1-negative (blue) for comparison of lifetime distribution between the two populations of CCPs. (D) Number of CCPs identified as NECAP1-positive or NECAP1-negative in control cells, cells treated with LP inhibitor (see [E] and [F]), pan-phosphatase inhibitor calyculin A (see Figure S5), and μ2^NAK−^ RPE cells. Values are mean ± SD, n= 18. (E and F) CCPs detected in RPE cells expressing EGFP-CLCa and NECAP1-mRuby2 and treated with LP inhibitor were grouped into cohorts with a specific range of lifetimes, and corresponding intensity traces of EGFP-CLCa and NECAP1-mRuby2 were plotted as a function of the time as in panels (A) and (B). (G and H) Scatterplots with values of density of persistent structures (G) and average lifetime of the 95th percentile (H) for cells treated with control siRNA (blue) and NECAP1 + NECAP2 depleting siRNAs (green). Lines represent mean and SD for each group, n = 20.

The recruitment profile of NECAP1-mRuby2 was analyzed by dual-color live-cell TIRFM (Figures 5A and 5B; Video S1). NECAP1-mRuby2 was present in 65% of CCPs. In these CCPs, the recruitment profile of NECAP1-mRuby2 paralleled that of the EGFP-CLCa as the CCP grows (Figure 5A). Most NECAP-positive CCPs have lifetimes over 30s (Figure 5C), whereas NECAP-negative CCPs are predominantly short lived. This distinct separation of lifetime distributions between NECAP-negative and NECAP-positive CCPs is similar to that previously described for dynamin positive versus negative CCPs (Aguet et al., 2013). Strikingly, the clathrin recruitment profile changed shape in NECAP-negative CCPs, especially in lifetime cohorts <40–60 s, acquiring a significant plateau followed by an extremely rapid (<5 s) complete loss in the EGFP-CLCa signal (Figure 5B). The short lifetimes of NECAP1 negative CCPs together with their distinct EGFP-CLCa intensity profiles may be indicative of a rapid disassembly process characteristic for labile clathrin coat rather than regulated membrane deformation into Ω structure and scission of the CCP.

Video S1. This TIRFM Video Shows a Time Lapse That Covers a Period of 10 Min and Visualizes Basal Surface of RPE Cells with EGFP-CLCa in Green and NECAP1-mRuby2 in Red, Related to Figure 5 Cells were treated with 0.1 DMSO (upper panel) or LP inhibitor (lower panel).

LP inhibitor treatment reduced the number of CCPs that recruit NECAP1-mRuby2 to 31% of all CCPs and the overall levels of NECAP1-mRuby2 declined by ∼50% (Figures 5D–5F). The clathrin-coated persistent structures in LP-treated cells (defined and analyzed in Figures 1G and 1H) are frequently deficient in NECAP recruitment (Figure S5A). The inhibition of protein phosphatase activity with Calyculin A increases the number of NECAP1-mRuby2-positive CCPs to ∼80% (Figure S5B). These experiments were repeated with NECAP2-mRuby2 and yielded very similar results (data not shown). The siRNA-mediated simultaneous depletion of both NECAP1 and 2 (Figure S5C) led to slower rates of CCP formation, an increase in the number of persistent structures (Figures 5G and 5H), and reduced rates of TfR uptake (Figure S5D). Interestingly, upon depletion of just a single NECAP isoform, we observed concomitant upregulation of the other NECAP isoform (Figure S5C). The compensatory upregulation of a single NECAP isoform may explain the mild effects on TfR internalization that we observed (Figure S5D, first two panels).

In conclusion, the quantity of NECAP1 in CCPs is positively correlated with the level of P-AP2. NECAPs are recruited in increasing amounts over time to the majority of CCPs throughout their life in a manner dependent on μ2T156 phosphorylation. Quantification of the immunofluorescence images (Figures S5E and S5F) and the resulting scatterplot show that there is also a positive association between the quantity of cargo (TfR) and NECAP in P-AP2 positive spots. This merely reflects that as the CCP grows it can accommodate more cargo since neither μ2T156-phosphorylation (Figure S3H) nor the additional presence of excess PHear domain (Figure S5G) affect AP2’s affinity for cargo. These data indicate that NECAP functions as a positive regulator of CCP growth and maturation by a mechanism that we subsequently investigate.

### Structure of the NECAP1 PHear Domain in Complex with T156-Phosphorylated μ2 Linker

**Figure 6:**
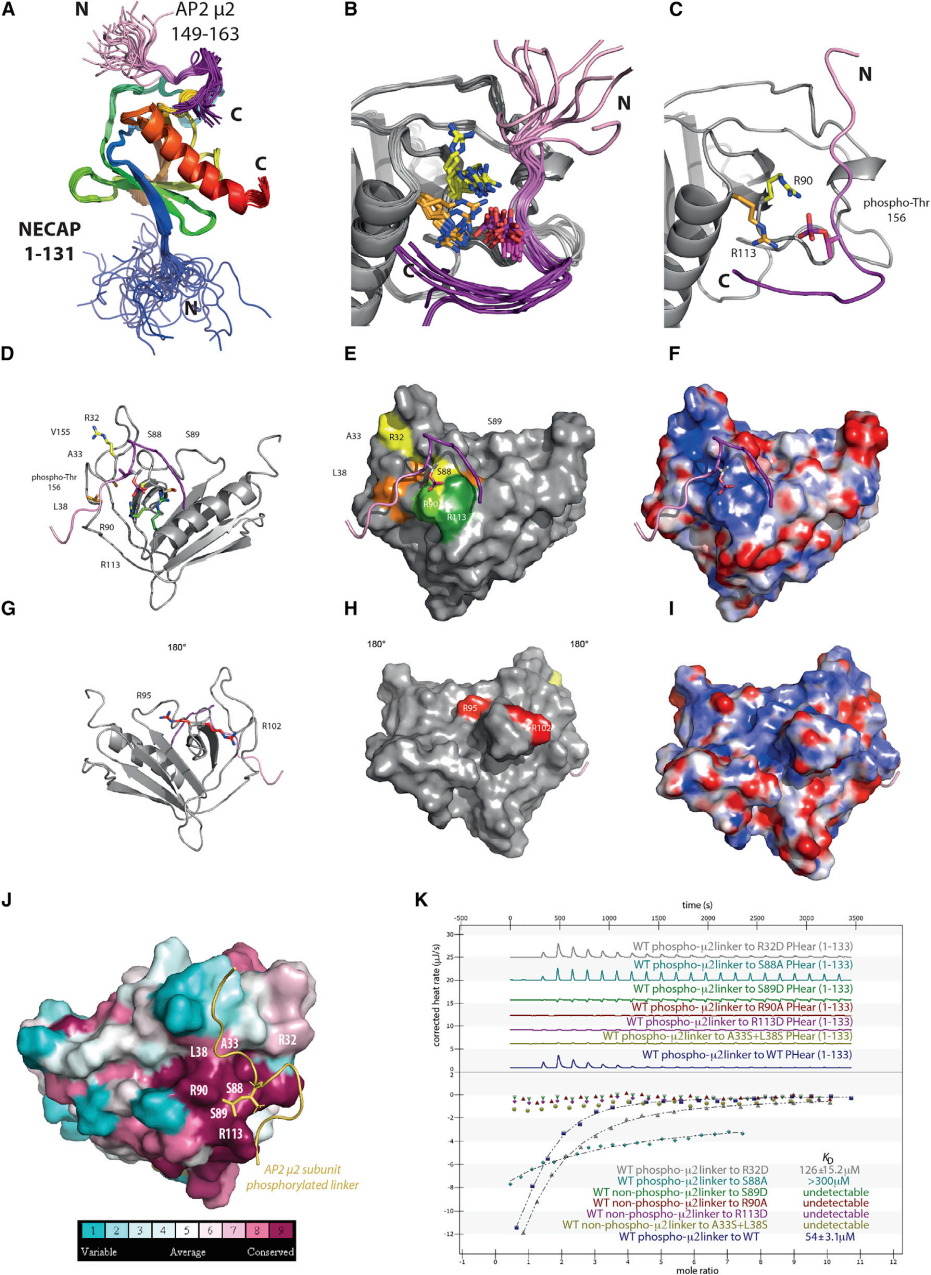
Mechanism of NECAP1 PHear Domain Binding to T156 Phosphorylated μ2 Linker (A) Family of the 30 lowest energy structures of the PHear domain of NECAP1 (colored blue N to red C) in complex with the T156 phosphorylated linker peptide (residues 149–163 colored pale to dark purple) of Cμ2. (B and C) Enlargement of best 10 structures (B) and single best structure (C) of the linker binding site, showing side chains of the strongest binding-abolition mutants Arg90Ala and Arg113Ala with carbons in light and dark green, respectively. Phosphate group is shown in purple (phosphorous) and red (oxygens). (D, E, G, and H) PHear:phospho μ2 peptide complex showing residues whose mutation strongly (light green, dark green, and orange) or mildly (yellow) affects the interaction as determined by ITC in (J). Mutations that do not affect NECAP binding but do affect amphiphysin and CALM binding via FxDxF motifs are shown in red and μ2 peptide in purples. (F and I) Electrostatic potential of PHear to highlight basic binding pockets of both binding sites colored from +10eV (blue) to −10eV (red). μ2 peptide in purples placed on after potential calculation. (J) PHear:phospho μ2 peptide complex colored according to the ConSurf conservation score (bottom) based on alignment of 150 sequences of 35%–95% sequence identity using the ConSurf algorithm. (H) Example ITC traces (top) and fitted curves with K_D_s (bottom) of binding of WT (dark blue) and mutants of NECAP1 PHear (cell) to WT μ2 linker phosphopeptide (syringe).

We probed the molecular details of the interaction by *de novo* determining the structures of the PHear domain and a complex between the PHear domain and the μ2 linker peptide SQITSQV(pT)GQIGWRR using multidimensional, heteronuclear NMR (Figures 6A and S6A–S6D; Table S4). The NECAP1 PHear domain is a member of the PH fold superfamily, having a seven stranded antiparallel β-sandwich core, one edge of which is packed against by the long C-terminal α-helix (Figure 6A) (Ritter et al., 2007). Comparison of the liganded and unliganded structures shows that peptide binding does not alter PHear domain conformation (Figures S6A–S6D). The phosphorylated μ2 peptide binds in a shallow groove lined with basic and hydrophobic residues. The main contacts are mediated by the side chains of R90 and R113, which are located within 3–4 Å of the phosphate group of the peptide throughout the ensemble of structures (Figures 6B–6D) and the side chain of S89, which contacts the backbone of R162 of the μ2 linker. The other significant peptide:domain interaction is made between V155 of the μ2 linker sitting in a shallow pocket formed by A33, L38, and T86, explaining why the mutant V155A phosphorylated μ2 peptide shows almost no binding to PHear (Figure S6E). The side chains responsible for the binding show very high conservation across 150 homologous sequences of 35%–95% identity (chosen across animals, plants, and fungi kingdoms), as determined with ConSurf (Ashkenazy et al., 2010) using HMMER search over UNIREF90 database with E value 0.0001 (Figure 6J). The rest of the peptide is disordered.

Structure-based mutations, which did not affect the domain’s fold as determined by circular dichroism and physical properties (data not shown), confirmed the mode of binding: mutation of R90A, R113D, and S89D and a double mutation of A33S/L38S abolished binding of the phosphorylated μ2 peptide, while mutation of S88A and R32D reduced binding (Figure 6K; Table S5). In contrast, mutation of R102A, which abolishes FxDxF binding (Ritter et al., 2007) and maps to a different patch on the PHear domain surface, had no significant effect on μ2 phosphopeptide binding (Figure S6E). There are two further indications that the binding sites for P-AP2 and Bin1/amphiphysin/RVS167 (BAR) domain proteins are independent, and hence can bind their ligands simultaneously: firstly the two sites are located on opposite faces of the PHear domain, and secondly a ∼20-fold molar excess of the amphiphysin-derived CSFFEDNFPE peptide has no obvious effect on the binding of the phosphorylated μ2 peptide to PHear in ITC or of Pcore to PHear in pull-downs (Figures S6E and S6F). N.b., the binding of FxDxF peptide to PHear in ITC gave data of insufficient quality to be reliably processed. To confirm this, mass spectrometry analyses of GST pull-downs from brain cytosol were carried out using wild-type (WT), R90A, R102A, and an R90A+R102A double mutant (Table S6). Amphiphysin1,2 and Bin2,1 were the most abundant proteins in a pull-down with NECAP1 1–133. Their capture was not affected by the R90A mutation, but capture massively decreased (>2^9^-fold) in the pull-down with R102A and the double mutant R90A/R102A. We note that AP2 capture was affected significantly, not only by the R90A mutation but also by the R102A mutation. This may be due to AP2 being at the hub of a large interaction network involving proteins binding via FxDxF motifs at the R102 site: in line with this, the levels of AP2 detected on pull-down with the double R90A/R102A mutant, decreased to the limits of detection.

### Point Mutation of R90 Inhibits Recruitment of NECAP to CCPs

NECAP1-mRuby2 WT or R90A were expressed at near endogenous levels in a background of siRNA-mediated depletion of endogenous NECAPs (Figures S6G and S6H). Native co-immunoprecipitation of AP2 demonstrated that the R90A NECAP1 showed ∼60% reduction in binding to P-AP2, and its expression caused ∼20% reduction in the TfR internalization rate as compared with re-expression of NECAP1-mRuby2 WT (Figures S6I–S6K). Further, we observed by TIRFM that NECAP1-R90A caused ∼50% reduction in recruitment to CCPs, which phenocopies reduced NECAP recruitment upon NAK inhibition.

### NECAP Links P-AP2 and the PX-BAR Proteins to Coordinate CCP Maturation

**Figure 7:**
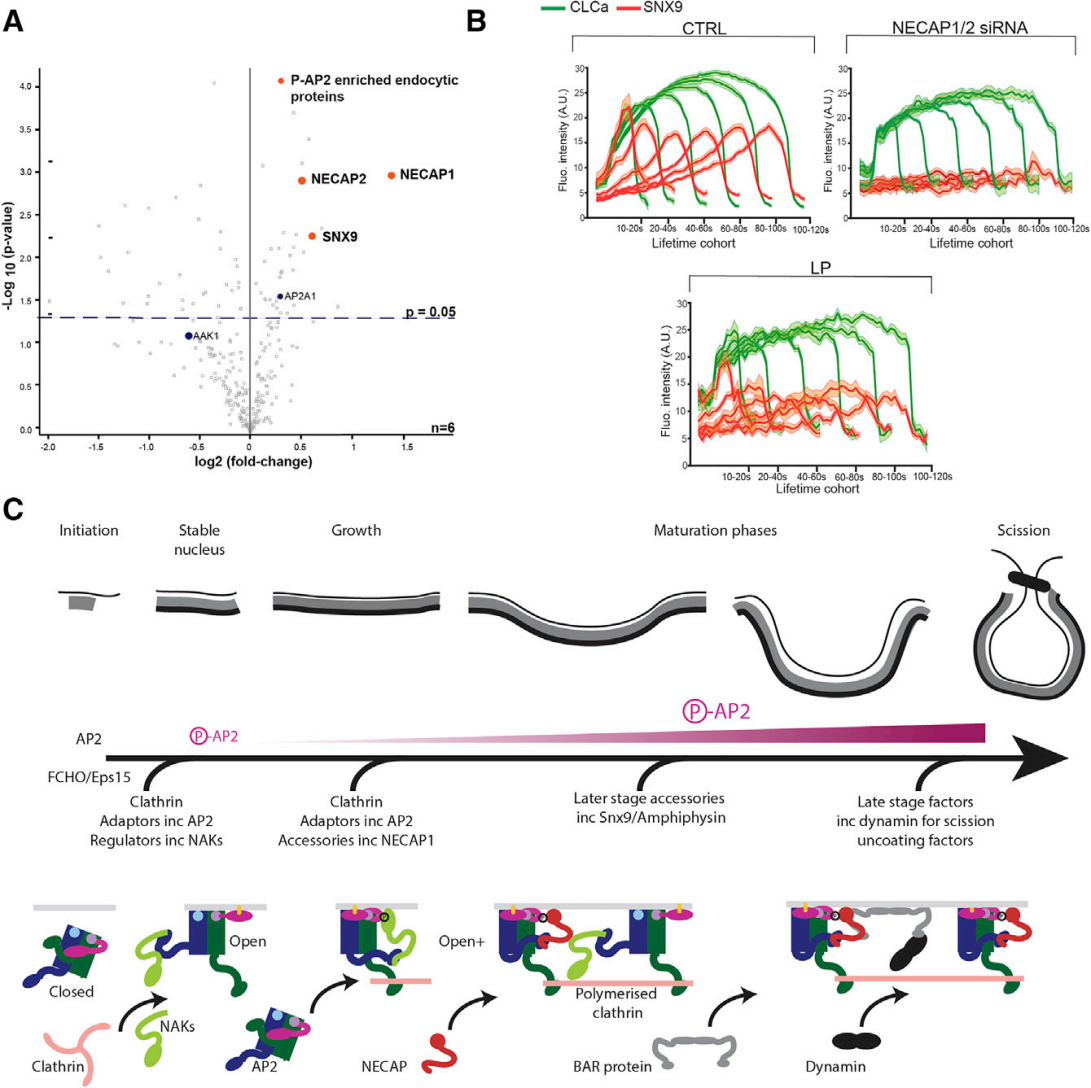
NECAP Recruits BAR Domain Proteins, Leading to CCV Scission (A) Analysis of the P-AP2 interactome. Volcano plot obtained from SILAC-based quantitative proteomics analysis of proteins that co-immunoprecipitated with AP2 in lysates obtained from control RPE cells treated with DMSO or cells treated with LP. Red dots represent proteins exhibiting significant (p < 0.05) fold enrichment in the P-AP2 interactome. n = 6. (B) Averaged CCP intensity traces for EGFP-CLCa (green) and SNX9 (red) from control cells (top left) and cells treated with NECAP1/2 siRNA (top right), and LP (bottom) are shown as mean ± SE (shaded areas) per lifetime cohort. (C) Schematic model of CCP maturation leading to CCV scission. The evolving CCP structure and the phases it passes through are shown at the top. The middle represents the degree of μ2T156 phosphorylation and cartoons of the individual components and their recruitment is shown below.

To quantitatively assess the changes in the AP2 interactome upon its phosphorylation, we used Stable Isotope Labeling with Amino acids in Cells (SILAC)-based proteomics combined with native co-immunoprecipitation of AP2. Since the NECAP PHear domain binds P-AP2 and can simultaneously bind endocytic accessory proteins via FXDXΦ motifs, this interaction survey should also identify binding partner(s) of NECAP. RPE cells were treated with LP inhibitor, while control cells were treated with just DMSO. NECAP1 and NECAP2, as expected but also the PX-BAR protein SNX9, underwent significant and reproducible depletion upon the inhibition of μ2T156 phosphorylation (Figure 7A). ITC measurements revealed that SNX9 can interact directly with NECAP via an FLDSL sequence in the unstructured linker that joins its SH3 and PXBAR domain, albeit with only moderately low affinity (Figure S7A), and this interaction is also seen upon immunoprecipitation of NECAP1-mRuby2 from cells (Figure S7B). SNX9 is a late stage CCP component that coordinates steps leading to membrane remodeling, PIP interconversion, and vesicle scission (Lo et al., 2017) and is abundantly expressed in non-neuronal cells. A portion of the SNX9 linker adjacent to the accessible FLDSL sequence, containing the sequence DPWSAW has been implicated in autoinhibition of SNX9 functions through binding back to and inactivating the membrane binding of the SNX9 BAR domain (Lo et al., 2017). The SNX9:NECAP PHear interaction should facilitate or trigger SNX9 activation by sterically inhibiting the DPWSAW binding back, freeing it, and allowing it to bind to the AP2 α-appendage and/or clathrin W-box site (Lo et al., 2017, Miele et al., 2004) and maintain SNX9 in an active conformation.

We tested the hypothesis that SNX9 recruitment and activation in CCPs may be dependent on the presence of NECAPs and their association with P-AP2 using TIRFM (Figure 7B). SNX9 recruitment to CCPs was blocked in cells depleted for NECAP1, 2 (Figure 7B; Video S2), and upon LP treatment, SNX9 recruitment was reduced by 50% as shown in lifetime cohorts in Figure 7B. Further, in control cells SNX9 is recruited later than NECAP, and in the majority of CCPs, SNX9’s maximum recruitment rate occurs around the time of, or after, the peak of maximal NECAP recruitment (Figure S7C).

Video S2. This TIRFM Video Shows a Time Lapse That Covers a Period of 10 Min and Visualizes Basal Surface of RPE Cells with EGFP-CLCa in Green and SNX9-mRuby2 in Red, Related to Figure 7 Cells were treated with control non-targeting siRNA (upper panel) or depleted simultaneously for NECAP1 and NECAP2 (lower panel).

To confirm these data in other cell lines, we used NECAP1 1–133 as bait in “pull-down” experiments with cytosolic extracts of three non-neuronal cell lines (HeLa, RPE, and HepG2). Identification of the bound proteins by mass spectrometry (Table S7) revealed that CALM (a previously noted partner of NECAP PHear [Ritter et al., 2013]) and SNX9 were the two most abundant endocytic proteins bound. Taken together with their domain compositions, these data suggest that SNX9 in non-neuronal cells and Amphiphysin1,2/Bin2,1 in neuronal cells are equivalent NECAP1,2 binding partners whose recruitment to CCPs is driven by AP2 phosphorylation.

## Discussion

The formation of a new CCP can be initiated either *de novo* by a pioneer complex of AP2 assisted by Fcho1/2 and Eps15/R (Hollopeter et al., 2014, Ma et al., 2016) or on the edge of existing large flat clathrin-coated structures (Lampe et al., 2016). In the productive situation, the clathrin:adaptor coat grows and together are deformed to form an “Ω” structure (Heuser, 1980, Kukulski et al., 2012). The CCV proceeds to scission from the parent membrane and finally to coat removal, triggered by PtdIns4,5P_2_ hydrolysis and HSC70/Auxilin-mediated clathrin-cage disassembly (He et al., 2017). All CCPs should ideally incorporate cargo and proceed toward completion of CCV scission, however, it is necessary that an off-path route exists so that CCV formation can be aborted under unfavorable circumstances (e.g., a lack of cargo) (Mettlen et al., 2018). This would ensure that the process does not become stalled with CCV components trapped in partly formed, non-productive structures.

Here, we have presented data showing that μ2T156 p-AP2 appears early and increases throughout CCP formation and that μ2T156 phosphorylation should favor conformational change to an open+ active conformation. Since both open and open+ forms of AP2 permit clathrin binding by release of the β2 linker (Kelly et al., 2014), both can participate in stabilizing CCP nucleating structures by recruiting clathrin. Various assays show that μ2T156 phosphorylation does not have a major effect on cargo and membrane binding by AP2, which is supported by structures showing that cargoes bind to Pcore and core in mechanistically identical manners. μ2Thr156 phosphorylation favors AP2, adopting a previously uncharacterized, cargo-bound conformation (Figure S7E), and thus in the absence of AP2 phosphorylation in cells, the equilibrium between AP2 conformations may be shifted toward the closed form: this could potentially result in a subtle deficit in cargo accumulation in CCPs on NAK kinase inhibitor treatment.

We show that μ2T156 phosphorylation by NAK kinases increases AP2’s affinity for NECAPs. This drives NECAP recruitment into endocytic CCPs, which we show also starts early in and increases throughout a CCP’s life. In the mammalian system, NECAP recruitment to AP2 is robust due to avidity effects caused by simultaneous recognition of the WxxF motifs at the C terminus of NECAP by the AP2 α-appendage along with the binding of the NECAP PHear to the phosphorylated the μ2 linker. The latter interaction will also mask T156 from dephosphorylation (Figure S7D). Further strength of binding could be derived from a possible interaction of the NECAP “ex” fragment with the β2 subunit (Ritter et al., 2013). Avidity-effect-enhanced binding of NECAP to α-appendage will also compete off NAK kinase bound to P-AP2, principally by its own WxxF motif so that it can phosphorylate further AP2s. There is 20-fold less AAK1 than AP2 in mammalian cells (Itzhak et al., 2016). N.b., there is also 4 times less AAK1 than NECAP (Itzhak et al., 2016). The competitive binding between AAK1 and NECAP to AP2 is also captured by quantitative proteomics using SILAC (Figure 7A). This complexity, based on the co-operativity between multiple binding modes of NECAP to AP2, together with the competition for binding with other CME players increases the specificity and adds further regulatory constraints on CCP growth, especially in higher vertebrates.

NECAP PHear can bind late-stage components of the CCV machinery such as SNX9, amphiphysin1,2/BIN2,1, and CALM (this work and Ritter et al., 2007, Ritter et al., 2013), while simultaneously being bound to P-AP2. This is possible because their respective binding sites are on distinct, non-overlapping sites on the NECAP PHear. The PHear-binding site on P-AP2 will be adjacent to the membrane surface due to the position of its phosphorylated μ2 linker, and thus a multi-attachment point recruitment platform for these late-stage membrane-remodeling components will be created at their site of function. As a side note, the FXDXΦ binding platform of the NECAP PHear domain is not conserved in *C. elegans* and concomitantly the FXDXΦ motif is absent in the *C. elegans* orthologs of the BAR domain proteins.

The fastest recruitment rates of SNX9 and amphiphysin1,2/BIN2,1 occur at the late stage of CCPs formation (Taylor et al., 2011 and this work). The final stage of CCV genesis is the recruitment by these BAR domain proteins of dynamin (whence it can form a “collar” to drive CCV scission) and synaptojanin (responsible for PtdIns(4,5)P_2_ and PtdIns4P hydrolysis (Aguet et al., 2013, Antonny et al., 2016, He et al., 2017, Owen et al., 1998). Thus, recruitment of NECAPs ultimately drives the completion of CCV formation, and hence, NECAP is a positive factor in endocytic CCP formation in mammalian cells. Indeed, in line with our model, we see that disruption of this phosphorylation-induced NECAP-based recruitment or depletion of NECAP itself, leads to a slowing down of the rate of the late stages of CCP maturation and an increase in the number of persistent, diffraction-limited, presumably stalled endocytic structures. This is consistent with the observed decrease in CME efficiency. It is interesting to note that depletion of SNX9 or dynamin, which either bind NECAP directly (SNX9 via FXDXΦ motif) or indirectly (dynamin via SNX9/amphiphysin1,2/BIN2,1) phenocopies both these effects of NECAP depletion (Antonny et al., 2016, Lo et al., 2017). Such a model in which as the CCP protein coat grows through binding of clathrin adaptors via PtdIns4,5P_2_, more cargo is incorporated by AP2, which in turn becomes progressively more phosphorylated by NAK to efficiently recruit NECAP and SNX9 and finally dynamin, is depicted in Figure 7C.

Thus, “feed-forward” control of the CCV formation pathway is defined through cargo-bound AP2 recruiting clathrin, which may in turn stimulate NAK-kinase-catalyzed phosphorylation of AP2 μ2T156. This then recruits NECAPs and subsequently their late-phase, membrane-remodeling, binding partners to the CCP. Hence, final membrane deformation and CCP scission will not occur efficiently until sufficient NECAP molecules and their bound late stage factors have been recruited. μ2T156 phosphorylation and NECAP recruitment can thus be considered to be at the center of the major decision that has to be “taken” of whether to proceed to completion of CCV formation with all the energy expenditure that would entail or to abort. Thus, AP2 phosphorylation followed by NECAP recruitment should be seen as a way of maximizing the chance of a CCV being “worth” forming, explaining why NAK kinase inhibition or depletion of NECAPs result in a reduction in CME efficiency rather than causing a complete block.

Additionally, our ITC data (Beacham et al., 2018) also suggest that by being able to bind closed P-AP2 in solution (Jackson et al., 2010), NECAPs could play a further, accessory role in CME by binding and stabilizing closed P-AP2 that has dissociated from a CCP membrane and so inhibit the P-AP2 from rebinding the membrane. To be consistent with our *in vivo* data, we surmise that NECAP would not be capable of significantly moving the equilibrium of open+ PAP2 to a closed conformation due to the open+ conformer being greatly stabilized by its simultaneous binding to multiple PtdIns4,5P_2_s and cargo. This function of NECAP could be especially useful at the CCP neck, where BAR proteins will have become concentrated and the presence of PtdIns4,5P_2_-binding dynamin spiral oligomers (Antonny et al., 2016) together will compete with AP2 for membrane PtdIns4,5P_2_ binding. Stabilizing unattached P-AP2 that has been locally competed off the neck region would facilitate neck scission by removing steric clashes between dynamin spirals and cargo-bound P-AP2. Such an additional role for NECAP is completely consistent with the data presented here.

In summary, the clathrin-activated NAK-kinase-catalyzed phosphorylation of the AP2 μ2linker stabilizes a cargo-binding competent conformation of AP2 in early and growth stages of CCV genesis. Throughout the lifetime of the CCP, P-AP2 is also pivotal to recruiting NECAP. NECAPs ability to simultaneously bind to P-AP2 and late stage components of CCV biogenesis means it can mediate the temporally appropriate recruitment of these latter proteins, which are necessary to convert mid-stage CCPs into mature CCVs. The interplay between the activating conformational changes in AP2, its phosphorylation, and the resulting recruitment of NECAPs confers direction and control on the process of endocytic CCV formation.

## STAR★Methods

### Key Resources Table

**Table:** 

REAGENT or RESOURCE	SOURCE	IDENTIFIER
**Antibodies**		

Anti-Phospho-AP2M1 (pT156) D4F3	Cell Signaling Technology	Cat#7399
Anti-Phospho-AP2M1 (pT156)	Cell Signaling Technology	Cat#3843
Anti-Phospho-AP2M1 (pT156) EPR4700	Abcam	Cat#ab109397
Anti-AP2M1	BD Biosciences	Cat#611350
Anti-NECAP1	Sigma-Aldrich	Cat#SAB1101485
Anti-AP2A	ThermoFisher	Cat#MA3-061
Anti-SNX9	Sigma-Aldrich	Cat#HPA031410
Anti-AP2M1	Abcam	Cat#ab75995

**Bacterial and Virus Strains**		

E coli BL21 DE3 pLysS	Invitrogen	N/A

**Biological Samples**		

Mouse brain	CECAD, Cologne, Germany	N/A
Pig brain	Local slaughterhouse, Cologne, Germany	N/A

**Chemicals, Peptides, and Recombinant Proteins**		

Peptide CD4Phos RM(Sphos)EIKRLLSE	Genscript	N/A
Peptide TGN38SPR CKVTRRPKASDYQRL	Genscript	N/A
Peptide CD4PhosSPR CHRRRQAERM(Sphos)QIKRLLSEK	Genscript	N/A
Peptide Amph FxDxΦ CSFFEDNFPE	Genscript	N/A
Peptide control ThrPhos IKIIDEK(Tphos)GVIEHE	Genscript	N/A
Amino acid ThrPhos (Tphos)	Sigma	N/A
Peptide μ2linker Phos SQITSQV(Tphos)GQIGWRR	Genscript	N/A
Peptide μ2linker SQITSQVTGQIGWRR	Genscript	N/A
Peptide μ2linker NAK- SQITSSVTAQIGWRR	Genscript	N/A
Inositol 1,2,3,4,5,6 hexakis phosphate	MERCK	CAS 14306-25-3 - Calbiochem
N15 NH_4_Cl	Goss Scientific	NLM-467 CAS 39466-62-1
C13 Glucose	Goss Scientific	CLM-1396 CAS 110187-42-3
1,2-Dipalmitoyl-sn-Glycero-3-Phosphoethanolamine-N-[4-(p-maleimidophenyl)butyramide]	Avanti, USA	N/A
pig-brain-derived phosphatidylcholine	Avanti, USA	N/A
pig-brain-derived phosphatidylethanolamine	Avanti, USA	N/A
pig-brain-derived PI(4,5)P_2_	Avanti, USA	N/A
Recombinant AP2 core coexpressed in E. coli	Collins et al., 2002	N/A
Recombinant phosphorylated AP2 core coexpressed in *E. coli*	Höning et al., 2005	N/A
Full-length NECAP variously tagged	This paper	N/A
Full-length NECAP PHear (1-133) variously tagged and mutants thereof	This paper	N/A
Peptide SNX9 FxDxΦ FLDSL	Genscript	N/A
AP2 μ2 subunit 122-435 and 160-435 variously tagged	This paper	N/A
NECAP PHear-Ex (1-178) variously tagged	This paper	N/A
NECAP Ex (133-178) variously tagged	This paper	N/A
Peptide TGN38 DYQRLN	Genscript	N/A
Peptide μ2linker V155A Phos SQITSQA(Tphos)GQIGWRR	Genscript	N/A
Peptide μ2linker V155A SQITSQATGQIGWRR	Genscript	N/A
Recombinant AP2 phosphorylated core with alpha ear coexpressed in E. coli	This paper	N/A
Recombinant AP2 core with alpha ear coexpressed in E. coli	This paper	N/A
Peptide Fluorscein-ASDYQRL	Sigma	N/A
Polymeric heparin (Av MW15kDa)	MERCK	CAS-2608411
L-LYSINE:2HCL (13C6, 99%; 15N2, 99%)	Cambridge Isotope Laboratories, Inc	
L-ARGININE:HCL (13C6, 99%; 15N4, 99%)	Cambridge Isotope Laboratories, Inc	

**Deposited Data**		

Pcore closed comformation	This paper	PDB ID: 6QH5
Pcore open+ conformation	This paper	PDB ID:6QH7
NMR NECAP PHear	This paper	PDB ID:
NMR NECAP PHear phosphorylated μ2linker complex	This paper	PDB ID:
Proteomics data: SILAC and pull-down experiments	This paper	PRIDE PXD013468
Core open+ conformation	This paper	PDB ID: 6QH6

**Experimental Models: Cell Lines**		

hTERT RPE-1	ATCC	ATCC CRL-4000

**Oligonucleotides**		

NECAP1 siRNA oligo: GCTTAAAAGGCCAGCGTCT	Dharmacon ON-TARGETplus Human siRNA series	https://dharmacon.horizondiscovery.com
NECAP1 ORF: siRNA oligo CGACCGAGTTGGAGTACGA	Dharmacon ON-TARGETplus Human siRNA series	https://dharmacon.horizondiscovery.com
NECAP2: siRNA oligo: GTAAATTGGCACCGTGTCA	Dharmacon ON-TARGETplus Human siRNA series	https://dharmacon.horizondiscovery.com
NECAP2 ORF: siRNA oligo: GTGGAGAGTGTGACGGATT	Dharmacon ON-TARGETplus Human siRNA series	https://dharmacon.horizondiscovery.com
μ2:siRNA oligo CCAAAGGCCAGUAAUGGAU	Dharmacon ON-TARGETplus Human siRNA series	https://dharmacon.horizondiscovery.com

**Recombinant DNA**		

Plasmid: pGEX4T3NECAP1(1-275) (full length)	This study	N/A
Plasmid: pGEX4T3NECAP1(1-133) WT and A33S, S88A, S89D, R90A, R90D, R95A, R102A, R113A point mutants therof	This study	N/A
Plasmid: pGEX4T2NECAP133-275	This study	N/A
Plasmid: pET28bHis_6_-NECAP1 (1-275)	This study	N/A
Plasmid: pET28bHis_6_-NECAP1 (1-133)	This study	N/A
Plasmid: pMWHis_6_β2t+μ2myc	Collins et al., 2002	N/A
Plasmid: pMWαtGST+σ2	Collins et al., 2002	N/A
Plasmid: pMWαGST+σ2	This study	N/A
Plasmid: pMWHis_6_β2t+μ2myc+AAK1(1-325)	Höning et al., 2005	N/A
Plasmid: pMIB6-μ2 WT	Kadlecova et al., 2017	N/A
Plasmid: μ2 NAK- and T156D mutants	This study	N/A
Plasmid: pMIB6mRuby2-Flag-GGGS-NECAP1	This study	N/A
Plasmid: pMIB6-mRuby2-Flag-GGGS-NECAP2	This study	N/A
Plasmid: pMIB6-mRuby2-Flag-GGGS-SNX9	This study	N/A

**Software and Algorithms**		

Topspin 3.1 and 3.2	Bruker GmbH, Karlsruhe, Germany	https://www.bruker.com/products/mr/nmr/nmr-software/software/topspin/overview.html
CCPN Analysis	Vranken et al., 2005	https://www.ccpn.ac.uk/v2-software/software/analysis
UNIO 2.8.1	Guerry et al., 2015	http://unio-nmr.fr
XPLOR-NIH 2.28	Schwieters et al., 2003	https://nmr.cit.nih.gov/xplor-nih
CCP4 v. 6 Containing Aimless, Refmac, Phaser, Prosmart, iMosflm,	Winn et al., 2011	www.ccp4.ac.uk
MATLAB R2016	MathWorks, Natick, MA, USA	https://www.mathworks.com/matlabcentral

### Lead Contact and Materials Availability

Further information and requests for resources and reagents should be directed to and will be fulfilled by the lead contact Dr Z Kadlecova (zk241@cam.ac.uk).

### Experimental Model and Subject Details

#### Cell Lines and Culture

Human retinal pigment epithelial cells (hTERT-RPE-1) were used as a parental cell line. It was obtained from ATCC and cultured in DMEM medium (31966-021, Thermo Fisher Scientific-Gibco) supplemented with 10% FBS (Thermo Fisher Scientific). All cells were cultured at 37°C under 5% CO2. Cell lines were routinely tested for absence of mycoplasma contamination with MycoAlert Mycoplasma Detection Kit (Lonza). HepG2 and HeLa S3 cells used for preparation of cytosolic extracts were also obtained from the ATCC and were cultured under the same conditions.

### Method Details

#### Bacterial Expression Constructs

The constructs for bacterial expression of AP2 pMWHis_6_β2t+μ2myc, pMWHis_6_β2t+μ2myc+AAK1(1-325), pMWαtGST+σ2, pMWαGST+σ2 were as described in (Höning et al., 2005).

The constructs for expressing AAK1 constructs, pET28bAAK1 (1-317) and (26-350) were made by cloning the appropriate length fragments of AAK1 into the NcoI and XhoI of pET28b.

The following constructs were created for bacterial expression of various forms of NECAP1. pET28bHis_6_-NECAP1 (1-275), pET28bHis_6_-NECAP1 (1-133) were both created by cloning appropriate length fragments of NECAP1 into pET28b using NdeI and NotI sites. pGEX4T3NECAP1(1-275), pGEX4T3NECAP1(1-133) and pGEX4T2NECAP133-275 were created by cloning appropriate fragments of NECAP1 into pGEX4T3 using EcoRI and NotI sites. Point mutant versions A33S, S88A, S89D, R90A, R90D, R95A, R102A, R113A NECAP1(1-133) were generated using standard pcr-based mutagenesis in pGEX4T3NECAP1(1-133).

To make a construct for expressing GST alone, termed pETHis6-GST, GST was cloned using XbaI and EcoRI sites into pETM30.

All constructs were verified by sequencing.

#### Protein Purification

Phosphorylated and non-phosphorylated AP2 heterotetramers were expressed and purified as previously described (Collins et al., 2002, Höning et al., 2005). Phosphorylation was achieved by co-expression with the kinase catalytic domain (residues 28-333 of the Homo sapiens AAK1) and estimated by mass spectrometry as >95% and occurring at the single site μ2 Thr156 (Collins et al., 2002, Höning et al., 2005).

NECAP1 GST-fusion proteins were expressed in E. coli BL21DE3 pLysS at 22°C for 16 hours. Cells were lysed and insoluble material removed by centrifugation. Proteins were bound to GST sepharose in bufferA (200 mM NaCl, 20 mM TRIS pH 7.4) and washed extensively with ∼100 volumes of bufferA and the proteins cleaved from GST by overnight incubation at 22°C with thrombin. Cleavage was halted by the addition of 0.1 mM AEBSF and the free proteins collected and concentrated to c. 20 mg/ml and further purified by S200 gel filtration in appropriate buffers. Full length NECAP1 was appended at its C-terminus with a His_6_ tag and this protein was additionally purified using NiNTA agarose to remove non-full-length material.

#### Protein Crystallization and Structure Determination

Crystals of Pcore in its locked/closed state were grown by hanging drop vapour diffusion of a solution containing 12.5 mg/ml protein and 0.5 mg/ml inositol hexakisphosphate against a reservoir containing 20% PEG 1000; 100 mM Na+/K+ phosphate buffer (pH 7.2), 200 mM NaCl, and 10 mM DTT. The crystals were mounted and cryo-protected in a solution containing: 25% glycerol, 20% PEG 1000; 100 mM Na+/K+ phosphate buffer (pH 7.2), 200 mM NaCl, and 10 mM DTT, prior to flash cooling by plunging them into liquid nitrogen. The crystals were of the space group P 3_1_ 2 1 with one molecule in the asymmetric unit cell of dimensions 121Å, 121Å, 258Å; 90, 90, 120 and diffracted at best to 2.6 Å at the Diamond Light Source beam line IO4-1. The data were integrated with Mosflm and scaled and merged in Aimless. The structure was solved using Phaser with the closed core structure (PDB ID 2VGL) as a search model and refined using alternating cycles of Refmac and manual rebuilding in Coot to the final R and Rfree of 0.230 and 0.259 respectively (Table S1). When overlaid, the Pcore and core structures exhibited RMSD of 0.56 over 1717 residues with loops missing in 2VGL also being missing in the Pcore equivalent.

Crystals of AP2 Pcore and core in the open+ state were grown by hanging drop vapour diffusion from a solution containing 12 mg/ml AP2 core or Pcore, 1mg/ml TGN38 YxxΦ-based cargo peptide DYQRLN, and 5 mg/ml functional variant dileucine cargo RM(P)SEIKRLLSE from CD4 against a reservoir containing 7.5% PVA, 9% 1-propanol, 90 mM Hepes pH 7.4, and 100 mM guanidine hydrochloride over a period of two days. Drops were set up with a 2:1 protein/precipitant ratio. The crystals were cryo-protected with 8% PVA, 12% 1-propanol, 90 mM Hepes pH 7.4, 100 mM guanidine hydrochloride, 20% glycerol, 1 mg/mL TGN38 peptide, and 5 mg/mL CD4 peptide before being plunged into liquid nitrogen. Data were collected at the Diamond Light Source beam lines IO4 and IO4-1, integrated in Mosflm and were of the space group P4_1_ 2_1_ 2 with unit cell dimensions of 182 Å, 182 Å, 211 Å; 90, 90, 90.

For the Pcore, data from five crystals diffracted to better than 4Å resolution but all were prone to radiation damage. Initially, each dataset was independently scaled and merged in Aimless and the structure was solved for several combinations of datasets with Phaser, using the open form (PDB ID 2XA7) as the search model. In the end, an “optimal” data selection was chosen by comparing the data from the five crystals with the calculated intensities from the initial model, as a function of image number: data from two crystals and from the later parts of two others were rejected as having too much radiation damage, leaving data from three crystals. Using this data set, the best molecular replacement solution was found using the bowl (α trunk, β2 trunk, Nμ2, σ2) from the open form and the Cμ2 domain from the same structure as independent search models (as opposed to the bowls and Cμ2 structure of other AP2 complexes). This final solution still exhibited very weak electron density for the first 80 to 100 amino acids of the N-terminal part of the β2 trunk although fully present as shown by complete rebinding of an aliquot of the crystallised Pcore to NiNTA resin through the His_6_ tag on the very N-terminus of β2 (data not shown). Various approaches were employed in an effort to locate these missing residues but all failed. Extensive refinement was carried out on the structure using Refmac. The initial refinement was attempted using rigid bodies with a hope that it would reveal the density for the N-terminal part of the β2 solenoid. As it did not, the β2 solenoid up to the residue 87 was removed, as the electron density around this region remained very weak. Prosmart was used to generate restraints for low-resolution refinement. Finally rounds of TLS refinement (using TLS groups previously determined for the open structure; Jackson et al., 2010), standard restrained refinement, and restrained refinement with the “bowl” restraints from the open structure and from Prosmart were all alternated with manual structure adjustment in between refinements in coot. Using this approach, the 3.4 Å structure of the AP2 Pcore in a new, open+ conformation was determined and refined to R=0.205 and Rfree=0.247 – the best statistics for an AP2 structure to date. DEN refinement and feature-enhanced maps were used with the hope of revealing more features in the final maps but both failed.

Crystals of the AP2 core in the open+ state diffracted significantly worse than those of the Pcore. The best dataset was collected from a single crystal diffracting to 6 Å at the Diamond Light Source beam line IO2. The data were integrated with Mosflm and scaled and merged in Aimless and the structure was solved with Phaser using the Pcore open+ structure as a search model. The validity of the obtained structure was confirmed by solving it again in Phaser using just the bowl (α trunk, β2 trunk, Nμ2, σ2) from the Pcore open+ form. The resulting solution showed clear electron density corresponding to the Cμ2 domain at the new position observed only in the open+ conformation. The validated full open+ core structure was then refined in Refmac to R=0.259 and Rfree=0.285. Crystallographic statistics of all three structures (Pcore closed, Pcore open+, and core open+) are listed in Table S1.

All programs used in crystallographic structure determinations were launched from and are part of CCP4 suite (Winn et al., 2011).

#### NMR Spectroscopy

##### NMR Sample Preparation

Samples for NMR were expressed in M9 minimal media supplemented with natural isotopic abundance glucose, vitamins, trace elements, antibiotics, and ^15^NH_4_Cl (for single-labelled samples) or ^13^C_6_ glucose vitamins, trace elements, antibiotics, and ^15^NH_4_Cl (for double-labelled samples). Following purification by gel filtration, samples were exchanged into 75mM sodium acetate pH 6.5 using a PD10 column (GE Healthcare) in accordance with the manufacturers instructions for the ‘gravity’ method.

NMR samples of NECAP 1-133 comprised 0.3-0.6mM ^15^N- or ^15^N,^13^C-labelled protein solutions in 70 mM ^2^H_3_ acetate buffer pH 7.0 in 95:5 H_2_O:^2^H_2_O or 99% ^2^H_2_O. In addition to the native NECAP residues 1-133, the sequence studied here carried a non-native N-terminal sequence GSPNS (here numbered -4-0). NMR samples of the complex comprised 0.3-0.4mM equimolar mixtures of ^15^N- or ^15^N,^13^C-labelled NECAP 1-133 and natural abundance μ2 149-163 in 70 mM ^2^H_3_ acetate buffer pH 7.0 in 95:5 H_2_O:^2^H_2_O or 99% ^2^H_2_O. NMR data were acquired using Bruker DRX600, AV-1 600, AV-3 800 spectrometers, each equipped with a cryogenically cooled triple resonance (^1^H/^15^N/^13^C) 5mm probe. Experiments were conducted at 25°C unless otherwise stated, and ^1^H chemical shifts were calibrated using sodium 3,3,3-trimethylsilylpropionate (TSP) as an external ^1^H reference; ^15^N and ^13^C chemical shifts were indirectly referenced to the ^1^H shifts using the ratio of gyromagnetic ratios. An essentially complete set of resonance assignments was made using the experiments described below.

For H_2_O samples of free protein (^15^N,^13^C-labelled NECAP 1-133), the following datasets were acquired: 2D datasets: [^15^N-^1^H] HSQC; [^13^C-^1^H] HSQC (separate experiments acquired for aliphatic and aromatic regions in ^13^C); constant-time [^13^C-^1^H] HSQC (separate experiments acquired for aliphatic and aromatic regions in ^13^C). 3D datasets: CBCANH; CBCA(CO)NH; HNCA; HBHA(CO)NH; [^1^H-^13^C-^1^H] HCCH-COSY; [^13^C-^13^C-^1^H] HCCH-TOCSY; ^15^N NOESY-HSQC (t_m_ = 150 ms); ^13^C NOESY-HSQC (t_m_ = 150 ms; separate experiments acquired for aliphatic and aromatic regions in ^13^C).

For H_2_O samples of complex containing 1:1 ^15^N,^13^C-labelled NECAP 1-133 and natural abundance μ2 149-163, the following datasets were acquired: 2D datasets: [^15^N-^1^H] HSQC; [^13^C-^1^H] HSQC (separate experiments acquired for aliphatic and aromatic regions in ^13^C); [^1^H-^1^H] filtered NOESY (t_m_ = 150ms) with filter elements set to reject protons coupled to ^13^C or ^15^N in F_1_ and to accept protons coupled to ^13^C or ^15^N in F_2_, so as to detect intermolecular cross-peaks; [^1^H-^1^H] filtered NOESY (t_m_ = 50 ms, 150ms and 200 ms) and [^1^H-^1^H] filtered TOCSY (t_m_ = 40 ms and 80 ms), all with filter elements set to reject protons coupled to ^13^C or ^15^N both in F_1_ and in F_2_, so as to detect intramolecular cross-peaks within μ2149-163. 3D datasets: HBHA(CO)NH; HNCACB; ^15^N NOESY-HSQC (t_m_ = 150 ms); ^13^C NOESY-HSQC (t_m_ = 150 ms; separate experiments acquired for aliphatic and aromatic regions in ^13^C).

For ^2^H_2_O samples of complex containing 1:1 ^15^N,^13^C-labelled NECAP 1-133 and natural abundance μ2146-163, the following datasets were acquired: 2D datasets: [^13^C-^1^H] HSQC (separate experiments acquired for aliphatic and aromatic regions in ^13^C), constant-time [^13^C-^1^H] HSQC (separate experiments acquired for aliphatic and aromatic regions in ^13^C), [^1^H-^1^H] filtered NOESY (t_m_ = 150ms) with filter elements set to reject protons coupled to ^13^C or ^15^N in F_1_ and to accept protons coupled to ^13^C or ^15^N in F_2_, so as to detect intermolecular cross-peaks. 3D datasets: [^1^H-^13^C-^1^H] HCCH-COSY; [^13^C-^13^C-^1^H] HCCH-TOCSY; ^13^C NOESY-HSQC (t_m_ = 150 ms; separate experiments acquired for aliphatic and aromatic regions in ^13^C); ^13^C filtered NOESY-HSQC (t_m_ = 150 ms; separate experiments acquired for aliphatic and aromatic regions in ^13^C), with filter elements set to reject protons coupled to ^13^C or ^15^N in F_1_, so as to detect intermolecular cross-peaks.

All of the NOESY datasets used for structure calculations (see below) were acquired using pulse sequences modified to ensure equal RF heating in each case, e.g. for ^13^C experiments, a period of ^15^N decoupling equal in length to the acquisition period was applied at the beginning of the interscan delay, and for ^15^N experiments an equivalent period of ^13^C decoupling was similarly applied.

##### Shift Perturbation Analysis

Backbone amide chemical shift perturbation analyses were performed by recording [^15^N-^1^H] HSQC spectra at 25°C during addition of natural abundance μ2146-163 to ^15^N-labelled NECAP 1-133. Chemical shift perturbations were calculated according to Δδ = √((Δδ(^1^H))^2^ + (Δδ (^15^N) / 5.0)^2^).

##### Structure Calculations

Initial structures of free NECAP 1-133 and of NECAP 1-133:μ2146-163 complex were calculated using the program UNIO 2.8.1 (Guerry et al., 2015), for which the input comprises the respective protein sequences, the full resonance assignment and the following processed 3D NOESY spectrum files: ^15^N NOESY-HSQC (t_m_ = 150 ms) in H_2_O, ^13^C aliphatic region NOESY-HSQC (t_m_ = 150 ms) and ^13^C aromatic region NOESY-HSQC (t_m_ = 150 ms) in H_2_O. For the complex, ^13^C aliphatic region NOESY-HSQC (t_m_ = 150 ms) and ^13^C aromatic region NOESY-HSQC (t_m_ = 150 ms) spectra acquired in ^2^H_2_O were also used as input, and upper-limit distance restraint files for intermolecular NOEs and intra-peptide NOEs were created following careful manual analysis of all the NOESY spectra and were supplied as additional input data to the UNIO calculations. The standard UNIO protocol was employed that consisted of seven cycles of NOESY peak identification, NOE assignment and structure calculation (Guerry et al., 2015). Each cycle comprised automated NOESY peak picking with ATNOS (Herrmann et al., 2002b), use of the resulting lists of peak positions and intensities as input for automated CANDID NOE assignment (Herrmann et al., 2002a), and use of the final set of meaningful, non-redundant NOE distance restraints from CANDID as input for structure calculation using the simulated annealing routine of CYANA (Güntert et al., 1997). In each UNIO–ATNOS/CANDID cycle, the output consisted of an updated list of assigned NOE cross peaks for each input spectrum and a final set of meaningful upper limit distance restraints which constituted the input for the torsion angle dynamics algorithm of CYANA for three-dimensional (3D) structure calculation. In addition, torsion angle restraints for the backbone dihedral angles ϕ and ψ derived from all backbone chemical shifts were automatically generated by UNIO and added to the input for each cycle of structure calculation. During the first six UNIO–ATNOS/CANDID cycles, ambiguous distance restraints were used. For the final structure calculation in cycle 7, only distance restraints were retained by UNIO that could be unambiguously assigned based on the protein three-dimensional structure from cycle 6.

Fifty structures were next calculated using XPLOR-NIH (Schwieters et al., 2003). As input, these calculations used the set of NOE restraints generated by the final (seventh) cycle of UNIO, re-formatted for use in XPLOR-NIH. Since the XPLOR-NIH calculations employed r^-6^ summation for all groups of equivalent protons and non-stereospecifically assigned prochiral groups, and since no stereoassignments were made (and the assignment-swapping protocol within XPLOR-NIH for deriving stereoassignments indirectly during the structure calculation itself was not applied), all constraints involving protons within such groups were converted to group constraints (by using wildcards such as HB^∗^). All lower bounds were set to zero. Structures were calculated from polypeptide chains with randomized ϕ and ψ torsion angles using a two-stage simulated annealing protocol within the program XPLOR-NIH as follows: The first stage comprised Powell energy minimization (500 steps), dynamics at 1,000 K (20,000 steps), increase of the van der Waals force constant during tilting of the NOE potential function asymptote (4,000 steps), switching to a square-well NOE function then cooling to 300 K in 2,000 step cycles, and final Powell minimization (1,000 steps); the second stage comprised Powell minimization (500 steps), increasing dihedral force constant during 4,000 step cycles of dynamics at 1,000 K (with a strong van der Waals force constant and square-well NOE potential function), cooling to 300 K in 1,000 step cycles, and 2,000 steps of final Powell minimization.

All fifty structures calculated in XPLOR-NIH were finally subjected to a further stage of refinement using a full force field and an implicit water-solvent model as implemented in the program AMBER 11 (Case et al., 2005). Calculations comprised initial minimization (200 steps steepest descent then 1800 steps conjugate gradient), then two rounds of 20ps of simulated annealing (each comprising 5000x1fs-steps heating from 0 - 500K; 13000x1fs-steps cooling to 100K; 2000x1fs-steps cooling to 0K) and final minimization (200 steps steepest descent then 1800 steps conjugate gradient). The experimental distance restraints were applied throughout, and force constants for the distance restraints were increased linearly during the simulated annealing to a final value of 20 kcal mol-1Å-2. Implicit solvent representation using the generalized Born method was employed throughout (igb=1), and Langvin temperature control was used (ntt=3; gamma_ln=5). The 30 structures with lowest AMBER energies were selected for deposition.

There is a previously published NMR structure of NECAP 1-133 (pdb 1TQZ) (Ritter et al., 2007), but this differs somewhat from our own free protein structure (rmsd between the lowest energy structures of each ensemble over N, Cα, C' atoms for residues 7-131 is 4.82Å; Figures S6C and S6D); consequently we chose not to use 1TQZ in our comparison between the free and bound states of NECAP 1-133, so as to ensure that any differences identified were genuinely due to peptide binding. The topology of the secondary structural elements in 1TQZ is the same as for the structure of NECAP 1-133 reported here, but at a more detailed level differences include i) 1 TQZ has Ala at position 3 and Ile at position 55, whereas the samples used here have Thr 3 and Thr 55, ii) proline 78 is clearly in the *cis* conformation (Dd13C(b-g) = 8.82ppm; strong NOE cross peak seen from Tyr 77 Hα to Pro 78 Hα but not to Pro 78 Hd2/3) but is modelled as *trans* in 1TQZ; iii) 1TQZ results from analysis of samples in a different buffer (25mM sodium phosphate, 75mM NaCl, 0.5mM EDTA and 3mM DTT, pH 7.2), and iv) the number of medium and long-range NOE-derived restraints used during calculation of 1TQZ was rather low (148). Statistics from the structure calculations are presented in Table S4.

#### Liposome-Based Surface Plasmon Resonance Assays

##### Production of Liposomes

Peptides derived from the cytoplasmic domains of TGN 38 (CKVTRRPKASDYQRL) and phosphorylated CD4 (CHRRRQAERM(SP)QIKRLLSEK) were synthesized by GenScript, with an additional amino-terminal cysteine. These peptides were then conjugated to a 1,2-Dipalmitoyl-sn-Glycero-3-Phosphoethanolamine-N-[4-(p-maleimidophenyl)butyramide] (Avanti, USA) as described in (Höning et al., 2005, Jackson et al., 2010). Resulting lipopeptides were then used for synthesis of liposomes containing sorting signals.

The liposomes were synthesized by rehydration of a desired lipid film in 0.3 M sucrose followed by sedimentation, resuspension in 20 mM HEPES, pH 7.4, and extrusion through a 100 nm membrane. The “basic” liposomes contained 80% pig-brain-derived phosphatidylcholine and 20% phosphatidylethanolamine. Complex liposomes were prepared by substituting PC with 2% of a lipid-linked peptide and/or 4% of a phosphoinositide PI(4,5)P_2_.

##### Biosensor Experiments

All SPR experiments were run on BIAcore T200 machine (BIAcore AB, Sweden) with 10 mM Tris pH 8.7, 250 mM NaCl, 1 mM DTT used as a running buffer. Liposomes were deposited on a surface of an L1 biosensor to generate a “membrane mimic”. In order to do so, the system was equilibrated in the running buffer and the surface of the L1 sensor was primed with three 60 sec injections of 20 mM CHAPS. This was followed by sequential injections of the liposomes (at 0.25 mM final concentration) aiming to reach the target of 7000 RU in each flow cell. Subsequently, the loosely bound liposomes were removed by two pulse injections of 50 mM sodium hydroxide for 5 s each. The entire procedure was run at 10 μl/min. The affinities of the AP2 cores and Pcores to the immobilised liposomes were then measured at a flow rate of 30 μl/min. The proteins were injected at concentrations ranging from 50 nM to 50 μM for 90 sec (association) followed by a buffer flow for 510 sec (dissociation). The liposome surfaces were then regenerated by a 10 s pulse injection of 50 mM NaOH. In all analyses, the background signal from the protein binding to the basic liposomes (80% PC, 20% PE) measured at flow cell 1 was subtracted from the binding to the complex liposome species.

##### SPR Data Analysis

The kinetic analysis of Pcore and core binding to the liposomes was then carried out. None of the systems studied gave single exponential kinetics. The association and dissociation phases were therefore routinely analysed using a double exponential function, which returned reaction amplitudes for the fast and slow components of each phase. Reaction amplitudes for the fast and slow components of the dissociation phases were generally more reproducible than those for the association phases. These data were therefore used to obtain apparent equilibrium dissociation constants associated with the fast and slow components by fitting the change in amplitude with protein concentration using a simple 1:1 binding model. The results of the analysis are shown in Figure S3G and Table S2.

##### Isothermal Titration Microcalorimetry

Experiments were performed using a Nano ITC machine from TA Instruments. For ITC with peptides, PHear domains were gel filtered into 75 mM HEPES pH 7.4, 0.25 mM TCEP. Peptides were dissolved in the same buffer. PHear domains at concentrations between 0.10 and 0.15 M were placed in the cell at 12°C and peptides at concentrations between 1 and 5 mM (depending on a peptide) were titrated in with 20 injections of 2.43 μl each separated by 2.5 minutes. For ITC with WT PHear and Pcore, the proteins were gel filtered into 100 mM TRIS pH 8, 250 mM NaCl, 0.25 mM TCEP and experiments were carried out at 10°C with between 0.05 and 0.1 mM Pcore in the cell and with PHear domain between 2 and 3 mM being titrated in with 20 injections of 2.43 μl each separated by 2.5 minutes. A relevant syringe-solution-into-buffer blank was subtracted from all data and for constructs, which displayed measurable binding, a minimum of three independent runs that showed clear saturation of binding were used to calculate the mean K_D_ of the reaction, its stoichiometry (n), and their corresponding SEM values (see Table S5). Analysis of results and final figures were carried out using the NanoAnalyzeTM Software.

##### Fluorescence Anisotropy Measurements

A peptide encoding the TGN38 YxxΦ motif (sequence ASDYQRL) and modified at its N-terminus with fluorescein (Sigma-Genosys) was used in all binding experiments at a concentration of 50 nM. Various quantities of recombinant AP2 core or isolated C-μ2 were premixed with the fluorescent peptide and incubated for 10 minutes to allow the mixture to come to equilibrium. Protein / peptide mixtures were prepared in 10 mM Tris pH 8.7, 250 mM NaCl (for AP2 core / phosphorylated AP2 core) or 20 mM HEPES pH 7.4, 500 mM NaCl (for C-μ2). Fluorescence anisotropy measurements were carried out at 25^°^C with a Clariostar plate reader (BMG Labtech) using a 482/530 nm fluorescence polarisation module. Where used, polymeric heparin (porcine, from intestinal mucosa; activity 197.0 U/mg; mean MW 15kDa; Merck) was added to ∼500 μM concentration, and NECAP PHear domain was added to 200 μM. Mean fluorescence anisotropy values (3 or more measurements) were plotted against protein concentration and the curves fitted to a single-site binding model:

F = F_f_ + (F_b_ - F_f_).[L]/(K_d_ + [L])where F is the measured anisotropy in a mixture of free (f) and bound (b) fluorescent peptide with anisotropy of F_f_ and F_b_ respectively, [L] is the pseudo-first order concentration of ‘‘ligand’’ (in this case, C-μ2, AP2 or P-AP2) and K_d_ is the equilibrium dissociation constant. Curves were fitted with ProFit software using the Levenberg-Marquardt algorithm. Figures S3 and S5 shows data plots and curve fits.

In addition to C-μ2, and P-AP2 core pre-incubated with heparin or with a mixture of heparin and NECAP PHear domain, binding titrations were performed with AP2 core in the absence of heparin, resulting in only a slow linear increase in anisotropy as previously reported (Jackson et al., 2010), indicating a background of nonspecific binding (data not shown).

#### GST Pull-downs and Mass Spectrometry

Interaction partners of various recombinant fusion proteins were identified after incubation brain or tissue-culture cell lysates. For brain lysates, a single frozen mouse brain was thawed in 1 ml of buffer-T (25mM Tris pH 7.8, 140mM NaCl, 1mM DTT, 0.1mM AEBSF, phosphatase inhibitors, 0,1% NP40), cut to small pieces and slowly homogenized with a glass homogenizer. The final brain lysate was collected as the supernatant of three consecutive centrifugations (5 min/1000xg; 10 min/1,000xg and 30 min/100.000xg). Lysates of tissue-cultured cells were prepared from 15 confluent dishes (15cm ø). Cells were washed with PBS, scraped off the dishes and collected by centrifugation for 5min/500xg. The derived pellets were resuspended in 5x Vol buffer-T, incubated for 20min at 4°C and the final lysates were gained as the supernatants of two consecutive centrifugations (20min/21.000xg; 30min/100.000xg).

The following proteins were used as baits: GST (negative control), GST-Core, GST-Pcore, GST-NECAP1 PHear (residues 1-133) and mutants of thereof (R90A, R102A, R90A/R102A). Equal amounts (∼ 10 μg) of GSH-bead immobilized bait proteins were incubated with 1-3 mg of tissue or cell lysates in a total volume of 1 ml buffer-T and incubated 1h/4°C. Afterwards, beads with bound proteins were collected by centrifugation for 5min / 500xg. Lysates were removed and beads were processed for mass spectrometry-based identification of bound proteins after washing 2x with buffer-T and 2x buffer without detergent.

To test binding of full-length NECAP1 and NECAP1 PHear (1-133) to core and Pcore, they were expressed as N-terminally GST-tagged fusion proteins and immobilized to GSH-beads. An equivalent of 6μg was incubated with 20μg of core or Pcore for 15min at RT in buffer T. Subsequently, the beads were collected by centrifugation and washed 1x with buffer T and 1x with buffer T without detergent. Bead-bound proteins were resolved by SDS-PAGE, followed by western blotting and detection of the AP2 μ-subunit.

Binding of full-length NECAP1 and NECAP1 PHear (1-133) to various AP2 species was also tested in buffer B (250 mM NaCl, 20 mM TRIS pH 8, 0.1 % NP40, 1mM DTT, 0.1mM AEBSF,) following the same protocol but with proteins detected with Coomassie stain. In such experiments NECAP1 constructs were used as baits and AP2 core, AP2 Pcore, AP2 core or Pcore in presence of 0.5 mM or 5 mM CSFFEDNFPE peptide (derived from amphiphysin, containing the FxDxF motif), AP2 α-FLcore, and AP2 α-FLPcore were preys.

To assay the binding to phospho- / non-phospho versions of GST-μ2^(122-171)^ and GST-μ2^(149-163)^, NECAP were expressed and isolated as N-terminally 6His-tagged fusion proteins and immobilized to NiNTA beads. Details of incubation and washing were as above and binding was detected after SDS-PAGE and Coomassie staining.

Beads from pulldown experiments with bound proteins were resuspended in 25μl 50 mM Tris pH 7.5, 2M Urea, 1 mM DTT, 150 ng Trypsin and incubate for 30 min at RT. Then, 50 μl of 50 mM Tris pH 7.5, 2M Urea, 50 mM Chloracetamid were added followed by centrifugation (3 min / 500xg). This supernatant and a second supernatant derived from an additional washing with Chloracetamid containing buffer were combined and incubated overnight at RT. Digestion was then terminated by addition of 1μl TFA. The derived tryptic peptides were loaded onto activated stage tips (Sigma) and washed with 0.1% TFA and 80% acetonitrile, 0.1% TFA.

For LC/MS analysis, samples were analyzed on a Q-Exactive Plus mass spectrometer (Thermo Scientific) coupled to an EASY nLC 1000 UPLC (Thermo Scientific). Peptides were loaded with 0.1% formic acid in water (solvent A) onto a self-packed Poroshell column (50 cm × 75 μm, 2.7 μm sized EC120 C18, Agilent) and separated at a flow rate of 250 nL/min using the following gradient: 3 - 5% solvent B (0.1% formic acid in 80 % acetonitrile) within 1 min, 5-30% solvent B within 40 min, 30-50% solvent B within 8 min, followed by washing with 95 % solvent B for 10 min. The mass spectrometer was operated in data-dependent acquisition mode. The MS1 survey scan was acquired from 300-1750 m/z at a resolution of 70.000. The top 10 most abundant peptides were isolated within a 1.8 Th window and subjected to higher energy collisional dissociation fragmentation at a normalized collision energy of 27%. The automatic gain control target was set to 5e5 charges, allowing a maximum injection time of 110 ms. Product ions were detected in the Orbitrap at a resolution of 35.000. Precursors were dynamically excluded for 20 s.

All mass spectrometric raw data were processed with Maxquant (version 1.5.3.8) using default parameters. Briefly, MS2 spectra were searched against a FASTA database constructed from reviewed and unreviewed human sequences extracted from Uniprot (downloaded at: Sept 2018). A list of common contaminants was included in the searches. False discovery rates on protein and PSM level were estimated by the target-decoy approach to 1% (Protein FDR) and 1% (PSM FDR) respectively. The minimal peptide length was set to 7 amino acids and carbamidomethylation at cysteine residues was considered as a fixed modification. Oxidation (M) and Acetyl (Protein N-term) were included as variable modifications. The match-between runs option was enabled.

After mass spectrometry-based identification of proteins captured in a pulldown experiment, we first eliminated any hit (identified by the presence of one or more peptides) that was captured by GST, which served as a negative bait control. In a second step we deleted any low abundance protein (identified by the presence of two or less peptides) from the list, as we wished to focus only on the most prominent binding partners. For the identification of proteins binding specifically to Pcore, we eliminated any protein hit (more than a single peptide present) that bound to GST and/or Core. The remaining lists of proteins were ranked from the top downwards based on the iBAQ values of any hit. Results are summarised in Tables S3, S6, and S7.

#### μ2^NAK−^ and μ2^T156D^ Retroviral Constructs

The μ2^NAK−^ and μ2T156D sequences were generated using standard site-directed mutagenesis in the μ2 cDNA inserted in retroviral bicistronic IRES-BFP vector PMIB6 previously described (Kadlecova et al., 2017). μ2^NAK−^ sequence contained Q154 to S and G^157^ to A mutations.

#### mRuby2-Flag- NECAP1 and mRuby2-Flag- NECAP2 Retroviral Constructs

Gene sequences of human NECAP1 and NECAP2 (RefSeq NM_015509, and NM_018090), N-terminally fused with mRuby2-Flag-GGGS and flanked by Not1 and Sal1 were custom synthesized by IDT. Standard restriction cloning techniques were used for insertion into retroviral vector PMIB6.

#### mRuby2-Flag -SNX9

The cDNA of SNX9, with a sequence corresponding to NM_016224.4, was a gift of Folma Buss, CIMR. The SNX9 construct was made using Gibson Assembly Master Mix (E2611, New England BioLabs) to introduce mRuby2-Flag-GGGS at the N-terminus together with NotI and SalI restriction sites. The cDNA was inserted into PMIB6 using standard restriction cloning techniques.

#### Retrovirus Preparation

Retrovirus particles were generated by co-transfecting HEK 293T cells in the presence of polybrene (Merck) with 3 plasmids coding for Gag-Pol, VSV-G and the retroviral expression constructs in PMIB6. FACS was used to sort population of cells expressing fluorescent marker as described below.

#### RPE Cell Lines Stably Expressing μ2^NAK−^ and μ2^T156D^

After retrovirus infection, cohorts of cells expressing various levels of mutant μ2 subunits were sorted by FACS based on BFP intensity. The levels of expression of μ2 subunit within each stable cohort was validated by western blotting using the anti- μ2-antibody (AP50, BD Bioscience). The cell population with the expression levels closest to the endogenous μ2-adaptin was selected for further experiments. The absence of phosphorylation in μ2^NAK-^ cell line was tested with three different anti-μ2pT156 antibodies: rabbit monoclonal ab109397 (Abcam), rabbit monoclonal D4F3 (Cell Signalling), rabbit polyclonal #3843 (Cell Signalling).

#### RPE Cell Lines Stably Expressing mRuby2-Flag-NECAP1, mRuby2-flag- NECAP2 and mRuby2-flag-SNX9

Cohorts of RPE cells expressing several different levels of NECAP1, NECAP2 or SNX9 were sorted by FACS based on mRuby2 fluorescent intensity. Anti-NECAP antibody (SAB1101485, SIGMA) or anti-SNX9 antibody (Sigma, HPA031410) was used to detect expression levels closest to the endogenous protein by western blotting.

#### siRNA Transfection

siRNA mediated depletion of endogeneous μ2, NECAP1 or NECAP2 was carried out with 3 rounds of siRNA transfection at 12, 36, and 60h after plating. The experiments were performed on the fifth day. The target sequences for siRNAs were located at 3’UTR of the genes. The sequences were as following: NECAP1: GCTTAAAAGGCCAGCGTCT, NECAP2: GTAAATTGGCACCGTGTCA. Separate set of control experiments were carried out with NECAP siRNA targeting ORF for NECAP1: CGACCGAGTTGGAGTACGA and NECAP2: GTGGAGAGTGTGACGGATT. μ2 siRNA sequence was CCAAAGGCCAGUAAUGGAU. Targeting siRNA together with non- targeting control sequence was acquired from Dharmacon ON-TARGETplus Human siRNA series.

#### Triple Color Immunofluorescence Microscopy

Immunofluorescence of μ2pT156-AP2-CLCegfp, μ2pT156-CLCegfp-NECAP and AP2-CLCegfp-NECAP were prepared to decipher the quantity and distribution relationship between the stated components. Parental cell lines expressing CLCegfp alone or in combination with NECAP1-mRuby2 were grown overnight in 6-well plates on a glass cover slips at a quantity of 1x10^5^ cells. Cells were fixed with methanol at -20°C for 45s and incubated with 2% BSA for 30 min at 4°C. Cells were either incubated for 3h at 10μM of LP or in vehicle control (0.1% (v/v) DMSO) prior to the fixation. Cells were incubated with a rabbit monoclonal anti- μ2pT156 antibody (1:500, D4F3, Cell Signalling) or anti-AP2α antibody AP6 to stain for total AP2 at 4°C overnight. Cells were washed three times with PBS and further incubated with appropriate AlexaFluor labeled secondary antibodies (Life Technologies) for 1h. After three washes with PBS the cover slips were mounted in stainless-steel chamber and imaged in PBS on Zeiss Elyra microscope in TIRF mode at room temperature.

#### Dual Color Immunofluorescence with Transferrin Receptor

To assess the dependency of cargo colocalization to CCPs on μ2T156 phosphorylation, cells were pretreated with 10μM of LP or in vehicle control (0.1% (v/v) DMSO) for 3h. Cells were chilled at 4°C and pre-chilled solution of mouse monoclonal anti-Tfr antibody OKT9 (Bio-X-cell) containing also LP was added to the wells at a final concentration 4μg/mL. Cells were incubated at 4°C for 30min, fixed and permeabilized using PFA and 0.1% saponin for 20 min and processed for TIRFM as described above.

#### Quantification of Signal Distribution and Colocalization in Immunofluorescence Images

Immunofluorescence images of control cells, μ2^NAK−^ cells or cells exposed to NAK inhibitors were imaged under identical conditions, and post-acquisition image processing and analysis was also identical for all conditions to allow for direct comparison. The spatial relationship and the degree of colocalization between signals in dual-colour or triple-colour immunofluorescence images were analyzed using Matlab (MathWorks, Natick, MA, USA). The aim of the analysis was to characterize the recruitment of the CCP components (AP2, NECAP, CLCegfp and Tfr) in relation to AP2 phosphorylation. To that end we compared the results of the analysis for immunofluorescence images acquired with control cells and cells treated with NAK inhibitors or μ2^NAK−^ cells. We adapted CMEanalysis software (Aguet et al., 2013) and the analysis consisted of following steps, each described in detail below: object detection, intensity normalization and calculation of colocalization and distribution statistics.

##### Object Detection

EGFP-CLCa, AP2, P-AP2, TfR or NECAP signals were detected independently for the two or three fluorescence channels using the spot detection algorithms(Aguet et al., 2013, Zaritsky et al., 2017) (Aguet et al., 2013). Clathrin-coated structures (CCS) were assumed to be diffraction-limited, producing a signal that is well approximated by 2D Gaussian function in TIRF microscopy. Fluorescence signals were identified by numerically fitting a 2D Gaussian at candidate locations, and statistically testing the amplitude against the residual background noise estimated at each location. For the purpose of the statistical and quantitative analysis the main channel was denominated as a “master” and the dependent channels were denoted as “slaves”. The location of the detected signal in the master channel was used to determine the search radius for the detection of the overlapping signal in the dependent slave channel.

##### Intensity Normalization

CCP intensities from individual cells were normalized similarly as in CMEanalysis software. It consisted of selecting the median cumulative distribution of CCS intensities as a reference distribution and estimation of a scaling factor for each cell that minimizes the mean squared error between the scaled distribution and that of the reference.

#### Calculation of Colocalization and Distribution Statistics and Data Representation

##### Correlation of Signal Intensity for a Pair of CCP Components

Pearson correlation coefficient (PCC) (Zaritsky et al., 2017) for the paired intensity value between master channel and slave channel was calculated in all detections in respective master channel for each cell and the results are averaged from 3 independent experiments, each containing at least 20 cells and represented as mean value +/- SD. Single cell masks were manually annotated in each field of view and quantification of colocalization was performed as described in the previous paragraph.

##### Determining the Proportion of CCPs Containing Significant Signal

PCCs capture only linear relations of the paired intensity values (Zaritsky et al., 2017). To have a better understanding of the mutual relation of fluorescence intensities of paired signals, we calculated a function of proportions. This function describes evolution of the number of detections in master channel that contain significant signal in the slave channel. Significance of the signal was determined by a threshold, which was set identically for all measured conditions to allow for direct comparison between conditions.

Detections in master channel were divided into bins based on their fluorescence intensity. Non-overlapping intensity intervals of bins were estimated in such a way that the bins contain approximately the same number of detected spots. The proportion of spots in each bin that were above the given significance level in the slave channel was plotted as a function of the bin intensity. This function thus depicts the impact of the appearance of the signal in slave channel on the amount of fluorescence intensity in the master channel.

##### Single Colour and Dual Colour Live Cell TIRF Microscopy and Data Analysis

RPE cells expressing EGFP-CLCa and either NECAP1-mRuby2, NECAP2-mRuby2 or SNX9-mRuby2 were imaged using a Zeiss Elyra microscope, appended with a 100 × 1.49 NA Apo TIRF objective (Zeiss) and mounted on inverted microscope equipped with the Definite Focus System. Time-lapse image sequences from different cells were acquired at a frame rate of 0.5 frame/s using a sCMOS camera with 6.45 × 6.45 μm2 pixels (Photometrics). Similarly, near simultaneous 2-channel (488nm/561nm TIRF) videos were acquired at 0.5 frame/s. Cells in control experiments, μ2^NAK−^ cells, or cells exposed to NAK inhibitors were acquired under identical illumination and acquisition conditions. Quantitative analysis to distinguish bona fide CCPs and to measure CCP initiation rates, lifetime distributions, and density of static structures was performed using CMEAnalysis software (Aguet et al., 2013, Kadlecova et al., 2017, Loerke et al., 2011). On minimum 10 control and 10 treated cells consisted a single independent biological repeat and 3 biological repeats were conducted. Single cell masks were manually annotated in each field of view. The analysis reported in this work focuses on 2 types of clathrin structures found at the plasma membrane – bona fide CCPs and persistent clathrin coated structures. To identify bona fide CCPs (i.e. those structures undergoing successful growth after nucleation followed by maturation phase) we used method described in (Aguet et al., 2013) that is based on intensity thresholding of trajectories. The thresholding step discriminated between transient coat assemblies and bona fide CCP. The results of CCP lifetime dynamics are either presented in the form of lifetime distributions or average EGFP-CLCa (in green) and NECAP/SNX9 (in red) fluorescence intensity traces in lifetime cohorts. Persistent clathrin coated structures, are excluded from this lifetime analysis and analysed and represented separately. Persistent structures are defined as present throughout the entire acquisition. The significance of the results was determined with a one-tailed, two-sample t-test.

##### Calculation of Time of Maximum SNX9 Recruitment Rates and NECAP Maximum Intensity

NECAP-mRuby2 averaged intensity values for individual cohorts (sampled in 2s interval) were smoothed by Gaussian kernel (standard deviation 1.2) and a maximum was found with subsample precision using cubic interpolation. SNX9-mRuby2 maximum recruitment rate was computed in the same way, as a maximum of intensity differences (central difference was used). Averaged intensity values were calculated from videos acquired with minimum 10 cells in 2 separate experiments using SNX9-mRuby2 cells or NECAP1-mRuby2 RPE cells.

##### Transferrin Receptor Internalization

Transferrin receptor (Tfn) internalization at 37°C experiments were performed using anti-TfnR mAb (OKT9, Bio-X-Cell). Cell lines were grown overnight in 96-well plates at a density of 2.0 × 10^4^ cells/well. Plates were incubated with 4 μg/ml of OKT-9 at 37°C for the indicated time points. Cells were then immediately cooled down (4°C) to arrest internalization. Following a washing step to remove unbound ligand (1× PBS, 4°C), the remaining surface-bound ligand was removed from the cells by an acid wash step (5 ×, 0.2 M acetic acid, 0.2 M NaCl, pH 2.5, 4°C). Cells were washed with PBS and then fixed in 4% PFA in PBS for 20 min and further permeabilized with 0.1% Triton X-100/PBS for 10 min. Internalized OKT-9 was assessed using a goat anti-mouse HRP-conjugated antibody (Life Technologies), further developed with OPD (P1536, Sigma-Aldrich), and the reaction was stopped by using 2M H_2_SO_4_. The absorbance was read at 490 nm with BMG CLARIOstar Microplate Reader.

Internalization efficiency measured upon acute treatment with LP inhibitor was carried out in the presence of 10μM LP with cells pre-incubated with LP for 3h. Internalization experiments measured in μ2^NAK-^ cells, NECAP1-WT or NECAP1-R90A were carried out after siRNA mediated knockdown of endogenous protein (see above). Results of individual independent experiments (n=3) were pooled, averaged and presented as mean±SD, n=3. The statistical significance was analyzed by two-tailed, unpaired t-test.

##### Immunoprecipitation and Immunoblot Analyses

For immunoprecipitation experiments, cells were grown to 80% confluency in 15cm dishes. NAK kinase inhibitor 1, LP-935509 and NAK kinase inhibitor 2 (Cas No1093222-27-5) were reconstituted in DMSO as a stock solution at 10mM and used at 1/1000. Cells were incubated in fresh medium containing 10uM solution of either inhibitor or 0.1 v/v% DMSO for 3h. Media was removed from the dishes and cells were washed three times with ice cold PBS. Cells were scraped and then solubilized in 0.1% NP40 lysis buffer using 10x passage through a 25G needle, insoluble debris was removed by centrifugation of the lysates at 10,000 × *g* for 10 min. An equal amount of each protein lysate was incubated with anti-AP2α antibody AP6 conjugated to protein G-Sepharose beads overnight at 4°C. The immune complexes were analyzed by Western blot with rabbit anti-AP2- μ2 (Abcam, (ab75995)), anti-AP2α (#AC1-M11, Pierce), Anti AP2-μ2 pT156 D4F3 (Cell Signalling), SNX9 (Sigma, HPA031410), anti-NECAP 1/2 (SAB1101485, SIGMA).

##### SILAC

RPE cells were grown in custom-made DMEM (minus arginine and lysine; Invitrogen) supplemented with 10% dialyzed fetal calf serum (Invitrogen). l-arginine (84 μg/ml;) and l-lysine (146 μg/ml lysine; Sigma-Aldrich) were added to the “light” media, while and l-arginine 13C/15N and l-lysine 13C/15N (Cambridge Isotope Laboratory) to the “heavy” media at the same concentrations. The amino acid concentrations are based on the formula for normal DMEM (Invitrogen). Once prepared, the SILAC media was mixed and filtered through a 0.22-μm filter (Millipore) and stored at 4°C. RPE cells were passaged in SILAC media for at least 2 weeks (or 5 cells doublings) before harvesting to ensure complete incorporation of isotopic amino acids. Cells were harvested for heavy amino acid incorporation check and immunoprecipitation. Immunoprecipitation was carried out as described above using RPE cell lysate prepared from three 15cm dishes for each condition. 100uL of G-Sepharose beads conjugated to 500 ug of anti-AP2α antibody AP6 were used for each immunoprecipitation. After incubation at 4°C O/N beads were rapidly washed with lysis buffer at 4°C three times. We followed manufacturer's instructions for Trypsin-LysC mix digestion (Promega). Beads from both conditions were combined and incubated with aqueous solution of dithiothreitol at a final concentration of 5 mM at room temperature for 1h. Alkylation was carried out with with freshly thawed aqueous solution of iodoacetamide at a final concentration of 5 mM. The immunoprecipitated proteins were digested in 200 μL of buffer containing 4M urea, 50 mM Tris-HCl pH 7.5 and 5 μg/mL Trypsin and LysC mix at 37°C overnight. After the initial digestion, the samples were centrifuged for 5 min at 14 000 rpm and the supernatant was collected in a clean tube separate from the beads. Beads were washed twice in 100 μL of urea buffer and the supernatants were pooled. Digestion with fresh enzyme mix continued at 37°C for additional 12h at 2M urea buffer. In the next step samples were treated with trifluoroacetic acid (TFA) to stop digestion. We followed previously published protocol (Borner and Fielding, 2014) to desalt the tryptic peptides with stage tips with SDB–RPS filler material (Empore material 3M).

##### LC-MS Analysis

Peptides were eluted from SDB-RPS tips with 30 μl of 5 % (w/v) ammonium hydroxide in 80% acetonitrile (ACN) and dried a speed vac.

All samples were analyzed on a Q-Exactive Plus (Thermo Scientific) mass spectrometer that was coupled to an EASY nLC 1000 UPLC (Thermo Scientific). After resuspension in 10 μl 5% formic acid, 2% ACN the peptides were loaded with 8 μl solvent A (0.1% formic acid in water) onto an in-house packed analytical column (50 cm × 75 μm I.D., filled with 2.7 μm Poroshell EC120 C18, Agilent). Peptides were separated at a constant flow rate of 250 nL/min using the following gradient: 3 - 5% solvent B (0.1% formic acid in 80 % ACN) within 1 min, 5-30% solvent B within 40 min, 30-50% solvent B within 8 min, followed by washing with 95 % solvent B for 10 min. The mass spectrometer was operated in data-dependent acquisition mode.

The MS1 survey scan was acquired from 300-1750 m/z at a resolution of 70.000. The top 10 most abundant peptides were isolated within a 1.8 Th window and subjected to HCD fragmentation at normalized collision energy of 27%. The AGC target was set to 5e5 charges, allowing a maximum injection time of 110 ms. Product ions were detected in the Orbitrap at a resolution of 35.000. Precursors were dynamically excluded for 20 s.

##### Database Search

All mass spectrometric raw data were processed with Maxquant (version 1.5.3.8) using default parameters. Briefly, MS2 spectra were searched against a fasta database constructed from reviewed and unreviewed human sequences extracted from Uniprot (downloaded at: 16.6.2017). The Maxquant default list of common contaminants was included in the searches. False discovery rates on protein and PSM level were estimated by the target-decoy approach to 1% (Protein FDR) and 1% (PSM FDR) respectively. Trypsin was chosen as the digestion enzyme with a maximum of 2 missed cleavages. The minimal peptide length was set to 7 amino acids and carbamidomethylation at cysteine residues was considered as a fixed modification. Oxidation (M) and Acetyl (Protein N-term) were included as variable modifications. The match-between runs option was enabled. The LFQ algorithm implemented in Maxquant was used for label free quantification. For SILAC quantification (Arg10 and Lys8) multiplicity was set to 2 and the re-quantify option was enabled. Statistical analyses, data transformation, and filtering were performed in Perseus 1.6.1.1 and results were visualized using the same software. Identifications were filtered by removing reverse hits, matches based on modified peptides only, and common contaminants (‘standard filtering’).

### Quantification and Statistical Analysis

Quantification analysis for the all the data presented in this manuscript have already been detailed in the STAR Methods section above, associated with each experiment, as well as in the figure legends. In all instances, data were obtained from at least 3 independent replicates so that meaningful SDs could be quoted as defined in the figure legends. Datasets were tested for normal distribution and depending on outcome, populations were tested for significant differences using the two-tailed, unpaired t-test performed in GraphPad Prism6.

### Data and Code Availability

The data sets generated during this study are available as follows:

Structure coordinates have been deposited on the PDB with the accession codes 6QH5 closed Pcore, 6QH6 open+ core, 6QH7 open+ Pcore and NECAP PHear+μ2phosphopeptide 6RH6 and PHear 6RH5.

Mass spectrometry proteomics data have been deposited to the ProteomeXchange Consortium via the PRIDE partner repository with the dataset identifier PXD013468.

Live cell imaging datasets and immunofluorescence data sets are available from corresponding author ZK on request.

## Supplemental Information

Document S1. Figures S1–S7 and Tables S1, S2, S4, and S5

Tables S3. List of Pcore Interactants Identified by Mass Spectrometry, Related to Figure 4

Tables S6. List of Mouse Brain Lysate Interactants of NECAP1 PHear and Its Mutant Versions R90A, R102A, and R90A/R102A, Related to Figure 7

Tables S7. List of Identified NECAP1 PHear Interactants from Three Non-neuronal Cell Lysates, See Attached Information, Related to Figure S7

Document S2. Article plus Supplemental Information
