# Temporal Ordering in Endocytic Clathrin-Coated Vesicle Formation via AP2 Phosphorylation

**DOI:** 10.1016/j.devcel.2020.02.010

**Published:** 2020-03-09

**Authors:** Antoni G. Wrobel, Zuzana Kadlecova, Jan Kamenicky, Ji-Chun Yang, Torsten Herrmann, Bernard T. Kelly, Airlie J. McCoy, Philip R. Evans, Stephen Martin, Stefan Müller, Susanne Salomon, Filip Sroubek, David Neuhaus, Stefan Höning, David J. Owen

## Main Text

(Developmental Cell *50*, 494–508.e1–e11; August 19, 2019)

As a result of an author oversight in the originally published version of this article, the author Susanne Salomon was omitted from the author list. This error has now been corrected in the article online. The authors apologize for the error and any inconvenience that may have resulted.

