# Supplementary material for: Temporal Ordering in Endocytic Clathrin-Coated Vesicle Formation via AP2 Phosphorylation
Source: Dev Cell. 2019 Aug 19;50(4):494–508.e11. doi: 10.1016/j.devcel.2019.07.017 (PMC6706699; doi:10.1016/j.devcel.2019.07.017)
Supplement: Document S1. Figures S1–S7 and Tables S1, S2, S4, and S5 [file mmc1.pdf]

Developmental Cell, Volume *50*

Supplemental Information

Temporal Ordering in Endocytic Clathrin-Coated Vesicle Formation via AP2 Phosphorylation

Antoni G. Wrobel, Zuzana Kadlecova, Jan Kamenicky, Ji-Chun Yang, Torsten Herrmann, Bernard T. Kelly, Airlie J. McCoy, Philip R. Evans, Stephen Martin, Stefan Müller, Filip Sroubek, David Neuhaus, Stefan Honing, and David J. Owen

Supplementary Information

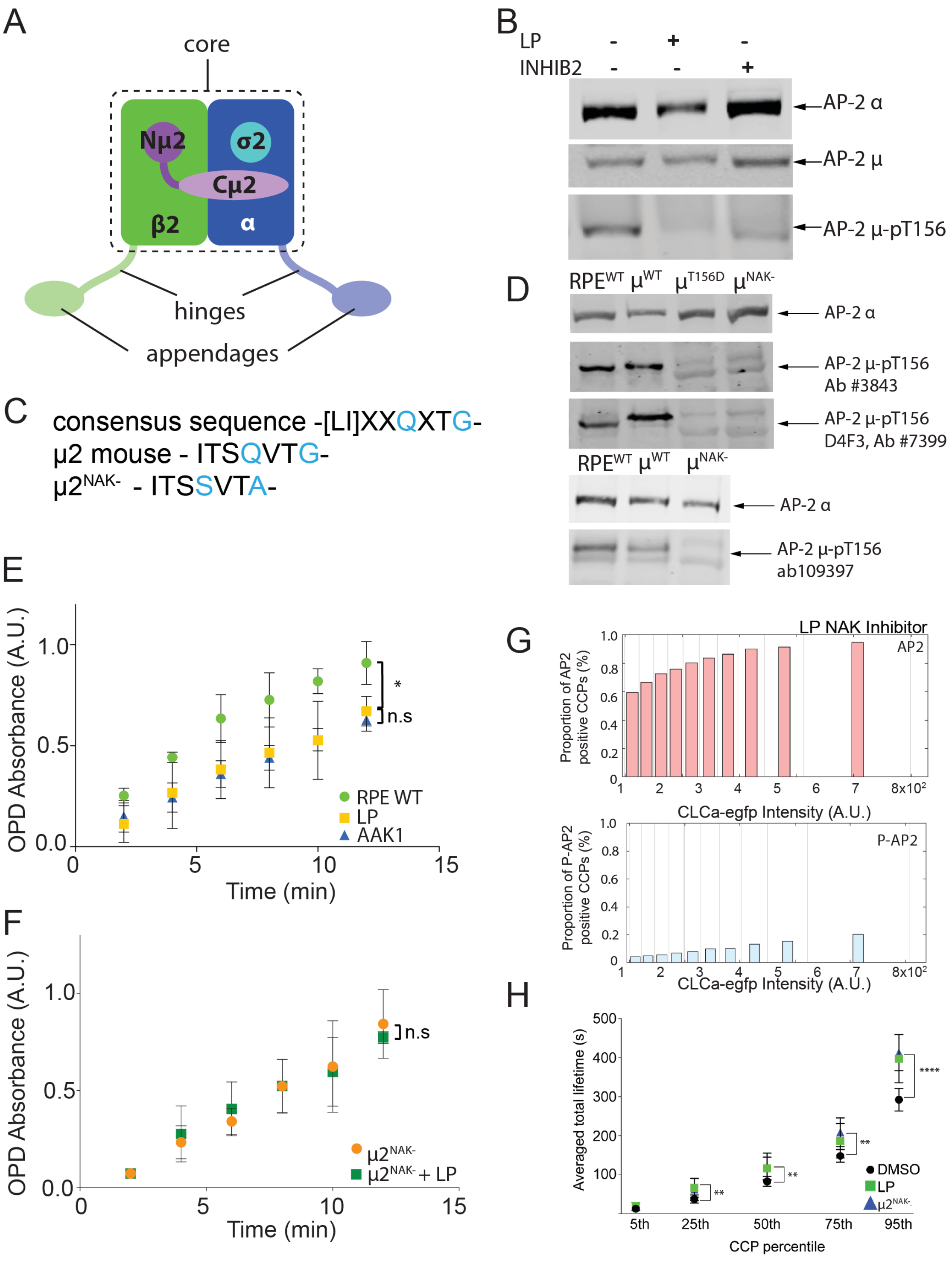

Figure S1 (related to Figure 1). μT156 phosphorylation can be acutely inhibited with specific small-molecular inhibitors.

A Schematic of heterotetrameric AP2 complex.
B Immunoblots of AP2 μ2 subunits using cell lysates obtained from RPE cells treated with vehicle control DMSO (at equal volume as inhibitor solution, i.e. 0,1% v/v), LP or inhibitor AAK1 (CAS 1093222-27-5) for 3h at 10uM.
C Conservative mutations in mouse μ2-adaptin sequence to generated $\mu2^{NAK-}$ construct. Mutations are targeting conserved residues to ablate NAK binding to μ2 adaptin.
D Immunoblots of AP2 $\mu2^{wt}$, $\mu2^{NAK-}$, $\mu2^{T156D}$ subunits expression in RPE cells. The variants of μ2-adaptins (that also harboured silent mutations to render them siRNA-resistant) were stably expressed in EGFP-CLCa expressing RPE cells by retrovirus infection. Cells expressing near-endogenous levels of the introduced subunits were selected by fluorescence-activated cell sorting, using an internal ribosome entry segment-expressed blue fluorescent protein (BFP). The selected population was used for further experiments. Prior to experiments cells were treated with μ2-adaptin siRNA to silence the endogenous μ2-adaptin. The absence of phosphorylation was verified with 3 different antibodies that specifically recognize the phosphorylated μ2 linker (polyclonal Ab #3843 and rabbit monoclonal D4F3 and Ab #7399 ab109397).
E Time course of TfR uptake in cells treated with DMSO, inhibitor AAK1 (CAS 1093222-27-5) or LP for 3h at 10uM. Data are represented as a mean with S.D. (n = 3).
F Time course of TfR uptake in $\mu2^{NAK-}$ and $\mu2^{NAK-}$ treated with LP for 3h at 10uM. Two-tailed Student's t-tests were used to assess statistical significance. $^{*}P < 0.05$.
G Bar graphs represent the quantification of the membrane distribution of AP2 (red) or P-AP2 (blue) in CCPs of LP treated cells. The bar height displays the proportion of CCPs (detected in EGFP-CLCa channel) that contain significant AP2 or P-AP2 signal in the same position plotted as a function of EGFP-CLCa signal (x-axis).
H Averaged total lifetime in 5th, 25th, 50th, 75th, 95th CCP percentile for $\mu2^{NAK-}$ cells and cells treated with LP in comparison to control cells, n = 18 cells, $^{**}P \leq 0.01$, $^{****}P \leq 0.0001$.

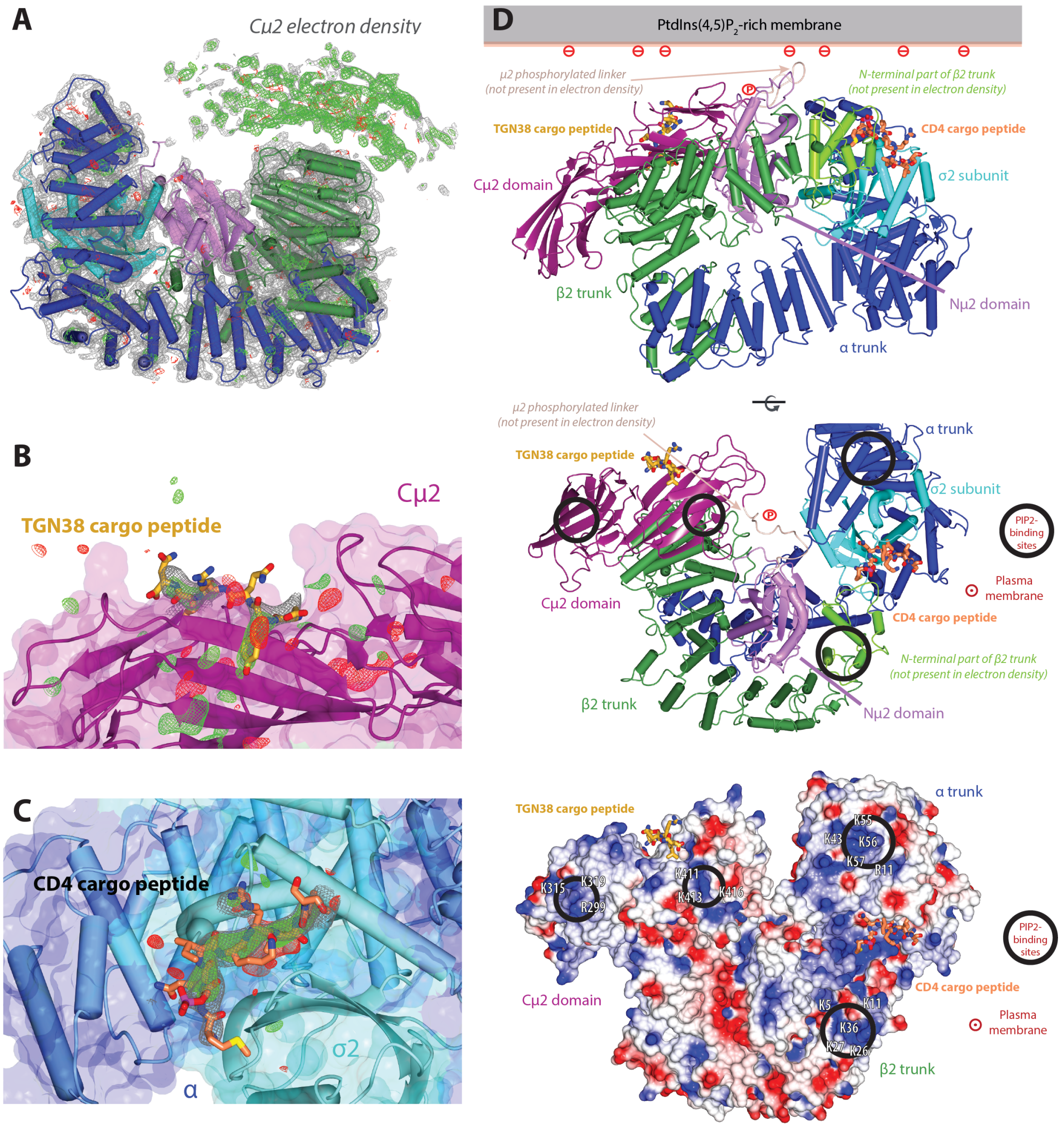

Figure S2 (related to Figure 2). A new open+ conformation for cargo-bound AP2 in both μ2T156phosphorylated and unphosphorylated forms

A-C Enlargements of functionally important regions with subunits coloured as in figure 2, unrefined 2mFo-DFc electron density shown in grey and difference density (always contoured at 3 sigma) in red (negative) and green (positive): **A** shows unambiguous Cμ2 positioning after first, partial molecular replacement solution (unrefined 2mFo-DFc map contoured at 1.5 sigma) **B** YxxΦ cargo peptide (unrefined 2mFo-DFc map clipped to 3Å around peptide, contoured at 1 sigma) and **C** dileucine cargo peptide (unrefined 2mFo-DFc map clipped to 3Å around peptide, contoured at 0.5 sigma).

D Highlighted important regions of the AP2 Pcore in the open+ conformation with subunits coloured as in main Figure 2. Views perpendicular to (top) and through (middle and bottom) the plasma membrane; previously established and proposed PtdIns4,5P_2–binding residues on the AP2 Pcore in the open+ conformation highlighted in the bottom panel (with all residues coloured according to their electrostatic potential: negatively-charged ones in red, positively in blue).

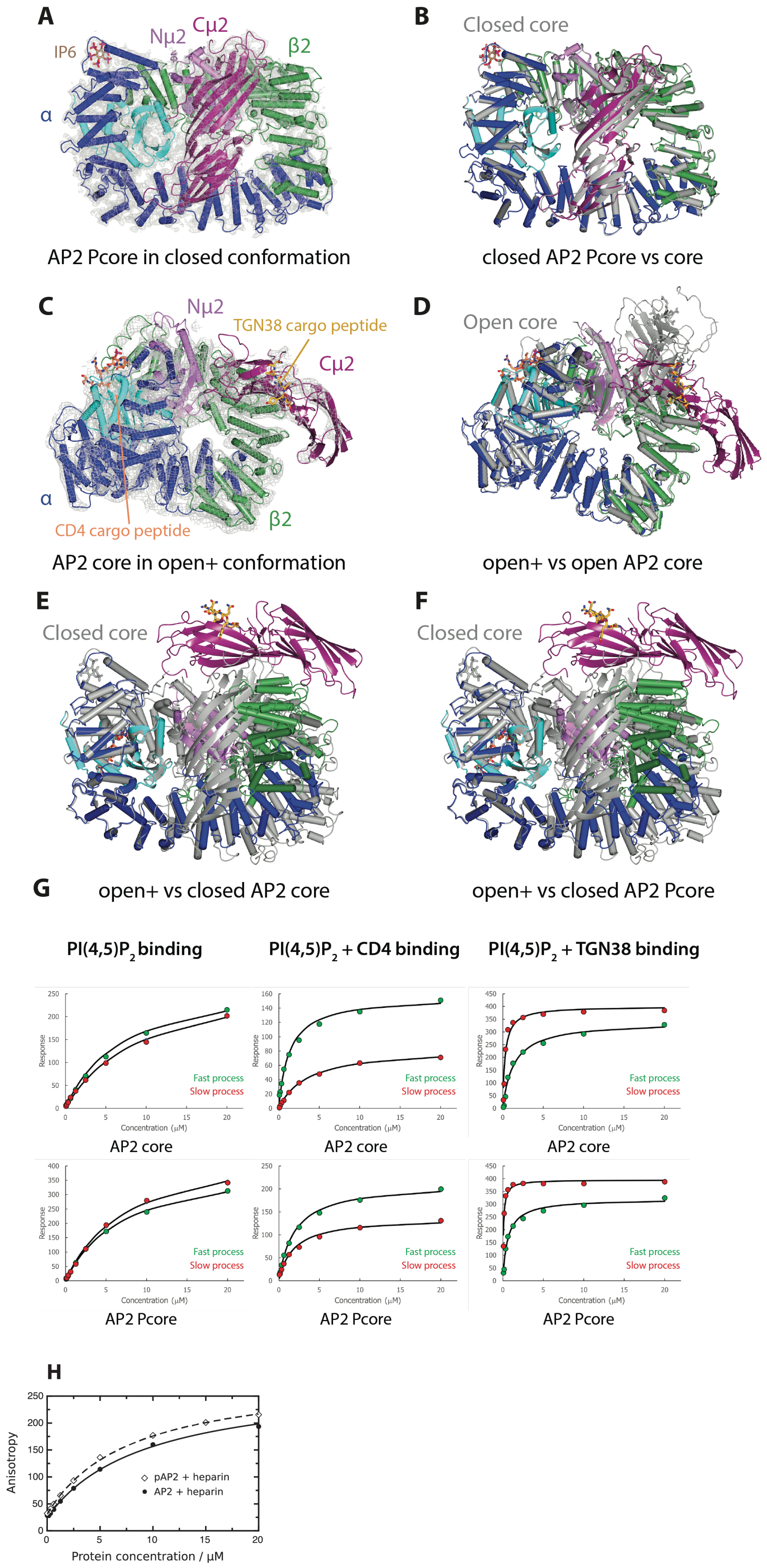
A
IP6
Nµ2
Cµ2
β2
α
AP2 Pcore in closed conformation
B
Closed core
closed AP2 Pcore vs core
C
Nµ2
TGN38 cargo peptide
Cµ2
α
β2
CD4 cargo peptide
AP2 core in open+ conformation
D
Open core
open+ vs open AP2 core
E
Closed core
F
Closed core
open+ vs closed AP2 core
open+ vs closed AP2 Pcore
G
PI(4,5)P2 binding
PI(4,5)P2 + CD4 binding
PI(4,5)P2 + TGN38 binding
Response
Concentration (µM)
Fast process
Slow process
AP2 core
AP2 Pcore
H
Anisotropy
Protein concentration / µM
pAP2 + heparin
AP2 + heparin

Figure S3 (related to Figure 3). Comparison between the open+ and other AP2 Pcore and core conformations.

A Structure of the AP2 Pcore in its closed conformation with refined 2mFo-DFc electron density (contoured at 1.5 sigma) and all the subunits coloured as in main Figure 2. PtdIns4,5P_2-analogue IP6 is shown in brown.
B. Comparison of the AP2 Pcore (coloured as before) and AP2 core (grey) in their closed conformations.
C. Structure of the AP2 core in its open+ conformation with refined 2mFo-DFc electron density (contoured at 0.4 sigma) and all the subunits coloured as in main Figure 2.
D Comparison of the open+ (coloured as before) and open (grey) conformations of the AP2 core.
E Comparison of the open+ (coloured as before) and closed (grey) conformations of the AP2 core.
F Comparison of the open+ (coloured as before) and closed (grey) conformations of the AP2 Pcore.
G Liposome-based SPR broken down into fast and slow processes (shown independently) for unphosphorylated and μ2T156-phosphorylated AP2 cores for PtdIns4,5P_2 membranes +/- YxxΦ or dileucine cargo measured at AP2 concentrations between 0 and 20 μM.
H Fluorescence anisotropy equilibrium binding curves. Comparison of P-AP2 and AP2 core activated by pre-incubation with heparin. Fluorescence anisotropy was measured for varying pseudo-first order concentrations of protein (as indicated) mixed with fluorescent YxxΦ peptide as described. Means of three or more measurements are plotted. Standard error on the mean is omitted for clarity but typically was in the region of 1% of the reported anisotropy. Curve fits are shown (solid line, AP2 with heparin; dashed line, P-AP2 with heparin). K_D values estimated from curve fits were similar (8.6μM ± 0.7μM for AP2, 7.8μM ± 0.3μM for P-AP2).

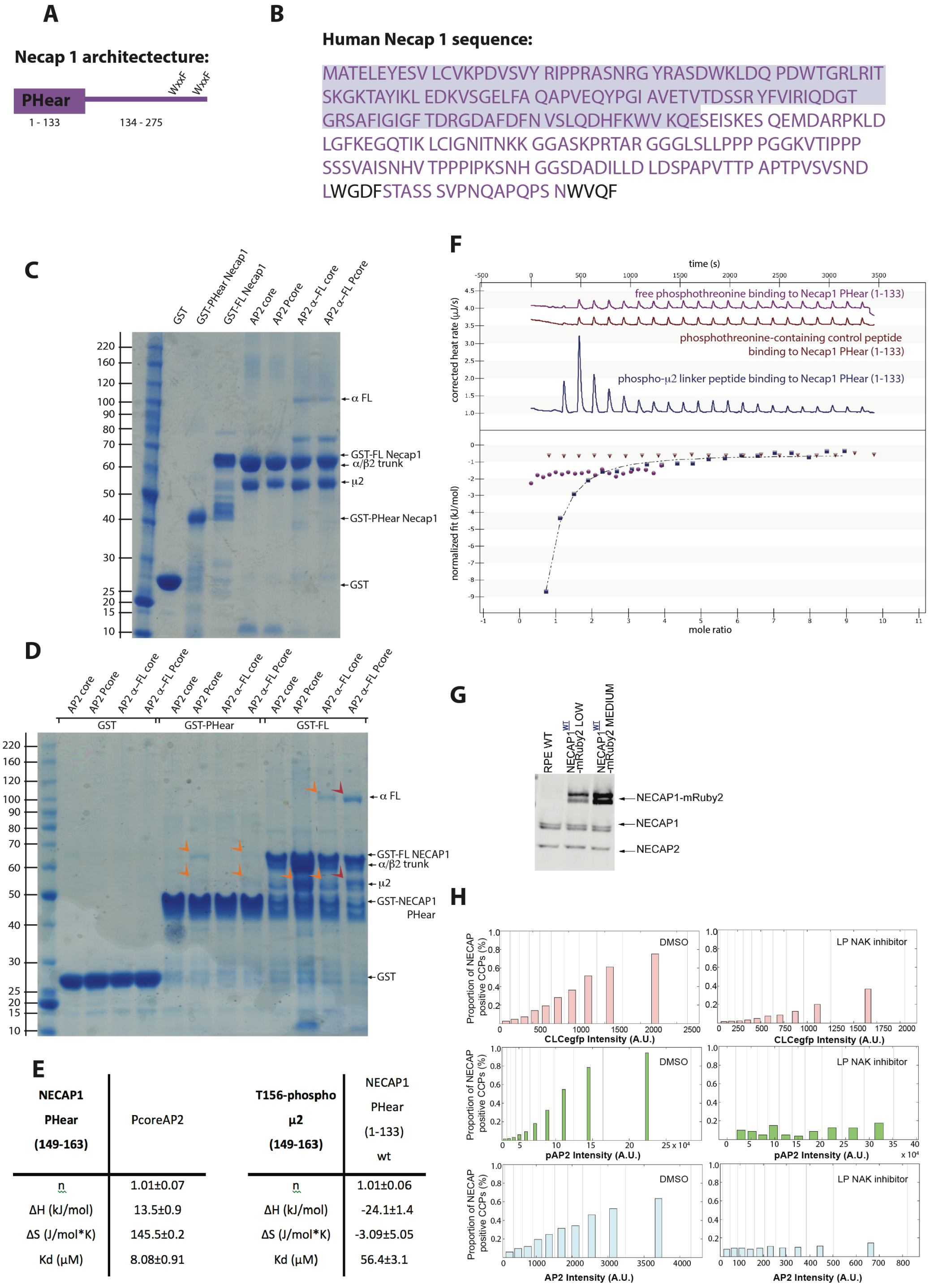

E

NECAP1 PHear (149-163)	PcoreAP2
n	1.01±0.07
ΔH (kJ/mol)	13.5±0.9
ΔS (J/mol*K)	145.5±0.2
Kd (μM)	8.08±0.91

T156-phospho μ2 (149-163)	NECAP1 PHear (1-133) wt
n	1.01±0.06
ΔH (kJ/mol)	-24.1±1.4
ΔS (J/mol*K)	-3.09±5.05
Kd (μM)	56.4±3.1

Figure S4 (related to Figure 4). NECAP interacts with P-AP2.

A and **B** Domain structure (A) and sequence (B) of Human NECAP1 with PHear domain boxed in purple and the WxxF motifs in black.
C Coomassie blue stained SDS PAGE gel of proteins used in E.

D 'GST pull downs' using the proteins indicated show that GST PHear will bind to phosphorylated (Pcore and α-FLPcore) but not unphosphorylated (core and α -FLcore) AP2 constructs whereas GST NECAP FL binds to Pcore, α-FLcore and α-FLPcore.

E. Thermodynamic parameters of the interactions between the PHear domain variants and phospho-μ2 linker peptides as well as Pcore determined by ITC.

F Example ITC traces (top) and fitted curves with K_Ds (bottom) of binding of wt NECAP1 PHear (cell) to (syringe): wt μ2 linker phosphopeptide (dark blue), an unrelated control threonine-phosphorylated peptide (red) or free phosphor-threonine (purple).

G. Immunoblot of RPE cells with ectopic expression of NECAP1-mRuby2. NECAP1 was N-terminally fused with fluorescent protein mRUBY2, FLAG-tag sequence and flexible linker sequence GGGGS. To achieve near-endogenous levels of stable expression of NECAP1-mRuby2 we used retroviral expression system with LTR as a weak promoter. For experiments we selected population of cells with low NECAP1 expression levels (middle lane).

H. Bar graphs represent the quantification of the membrane distribution of NECAP1 in RPE cells. Spot detection was carried out in master channel which was either EGFP-CLCa (red bar graph), P-AP2 (green bar graph) or total AP2 (blue bar graph). The bar height displays the proportion of spots (detected in master channel) that contain significant NECAP1-mRuby2 signal in the same pixel position. The data is plotted as a function of increasing intensity in the master channel (x-axis).

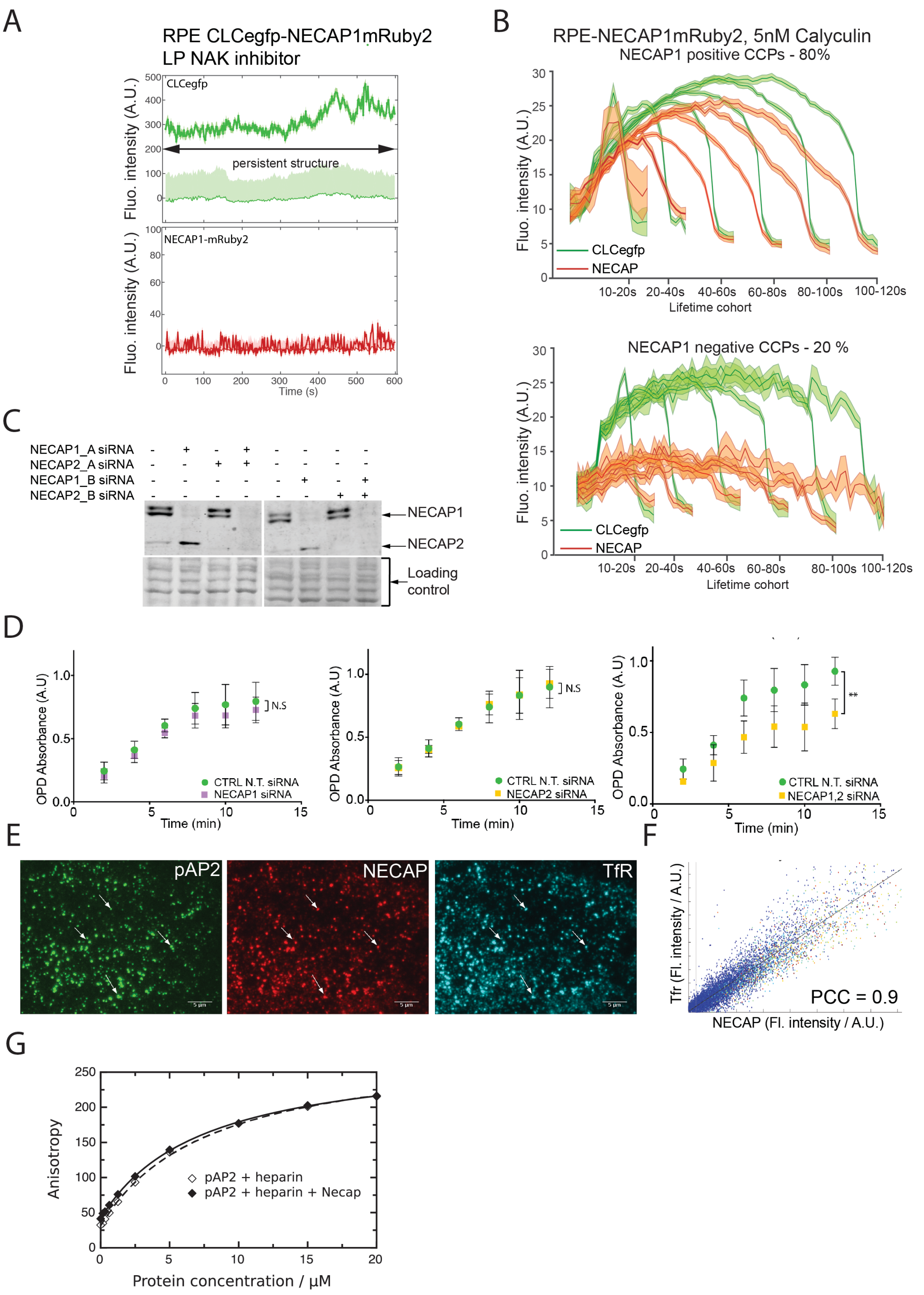

Figure S5 (related to Figure 5) µ2T156 phosphorylation causes NECAP recruitments to CCPs.

A Representative intensity trace of EGFP-CLCa (upper panel) and NECAP1-mRuby2 (lower panel) in a persistent trajectory spot in a cell treated with LP (3h, 10µM). Such a diffraction-limited clathrin coated structure is present throughout the entire 10min acquisition time.

B Plots show averaged intensity traces for EGFP-CLCa signal (green) and NECAP1 signal (red) imaged in cells treated with pan-S,T-phosphatase inhibitor Calyculin A (5nM, 30 min), n=20 cells. Traces are shown as mean ± SE (shaded areas) grouped by cohorts according to lifetimes. Upper panel shows intensity traces in NECAP1-mRuby2 positive CCPs and lower panel correspond to intensity traces in CCPs identified as NECAP1-mRuby2 negative.

C Immunoblots showing NECAP1 and NECAP2 depletion by 2 different sets of siRNAs targeting either ORF or 3'UTR.

Left panel-1st lane: RPE cells treated with non-targeting sirRNA (negative control), 2nd lane: NECAP1 depletion with Dharmacon ON-TARGETplus Human 3'UTR NECAP1 siRNA (GCUUAAAAGGCCAGCGUCU). 3rd lane: NECAP2 depletion with ON-TARGETplus Human 3'UTR NECAP2 siRNA (GUAAAUUGGCACCGUGUCA), 4th lane simultaneous NECAP1 and NECAP2 depletion using combination of both using 3'UTR siRNAs.

Right panel: -1st lane: RPE cells treated with non-targeting siRNA (negative control), 2nd lane: NECAP1 depletion with Dharmacon ON-TARGETplus Human ORF NECAP1 siRNA (CGACCGAGUUGGAGUACGA). 3rd lane: NECAP2 depletion with ON-TARGETplus Human ORF NECAP2 siRNA (GGUGGAGAGUGUGACGGAUU), 4th lane simultaneous NECAP1 and NECAP2 depletion using combination of above mentioned siRNA sequences targeting ORF of NECAP1 and NECAP2.

D Time course of transferrin receptor (TfR) internalization at 37°C in cells with NECAP1 depletion using ORF NECAP1 sirna (left panel), ORF NECAP2 siRNA (middle panel), and simultaneous NECAP1 and NECAP2 depletion using combination of siRNA sequence targeting ORF of NECAP1 and NECAP2. Data represent mean S.D., n = 3. Two-tailed Student's t-tests were used to assess statistical significance. **P < 0.01.

E Representative immunofluorescence images of RPE cells showing P-AP2, NECAPmRuby2 and TfR detection.

F The scatter plot and PCC value displays correlation between the intensity values of NECAP1-mRuby2 and NECAP in spots detected in P-AP2 channel. Plot represent data from n = 30 cells.

G Effect of PHear on affinity of heparin-activated Pcore for YxxΦ peptide Fluorescence anisotropy was measured for varying pseudo-first order concentrations of protein (as indicated) mixed with fluorescent YxxΦ peptide. Means of three or more measurements are plotted. Standard error on the mean is omitted for clarity but typically was in the region of 1% of the reported anisotropy. Curve fits are shown (solid line, P-AP2 with heparin and NECAP PHear domain; dashed line, P-AP2 with heparin only). K_D values estimated from curve fits were similar (7.8µM ± 0.3µM in the presence of NECAP, 7.8µM ± 0.4µM in the absence of NECAP.

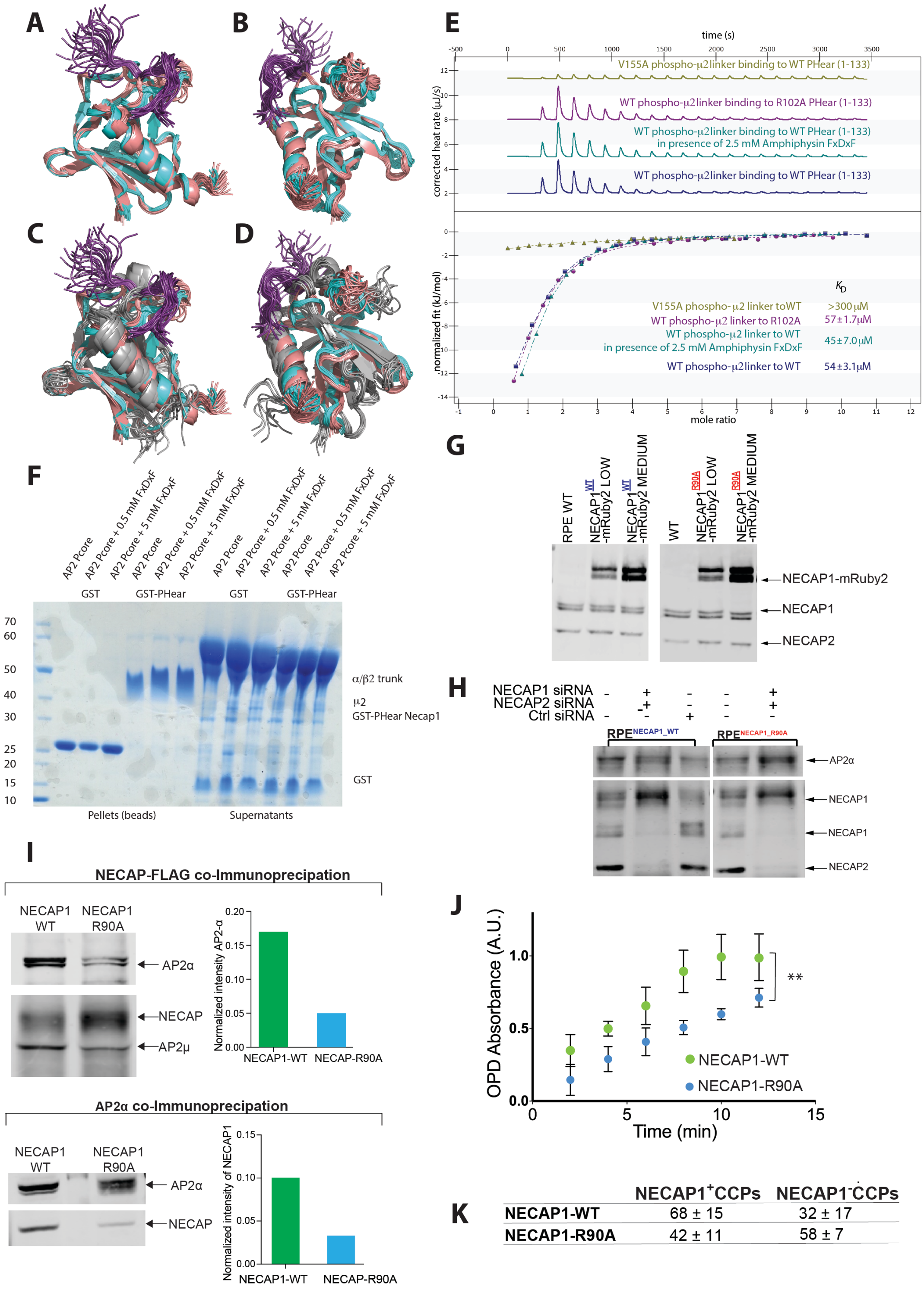

Figure S6 (related to Figure 6). Binding of NECAP1 PHear to μ2linker phosphorylated peptide

A-D Comparison of free and bound structures of PHear domain of NECAP1.

A and **B** Ensemble overlay of the NMR structures of NECAP1 PHear domain in the free state (salmon pink) and in complex with T156-phosphorylated **μ**2 linker (NECAP1 in cyan, peptide in purple). Fitting was calculated using the N, Cα and

C' atoms of residues 7-131 in each case. Views A and B are related by a 90° rotation about y-axis.
C and **D** Ensemble overlays as in A and B, but also including the previously published NMR structure 1TQZ (grey); fitting as before.
E Example ITC traces (top) and fitted curves with K_Ds (bottom) of binding of wt NECAP1 PHear (cell) to wt μ2 linker phosphopeptide (syringe) in the absence (dark blue) and presence in the cell of 2.5 mM FxDxF peptide from amphiphysin1/Bin2 (green). Binding of wt NECAP1 PHear (cell) to (syringe) V155A μ2 linker phosphopeptide (gold); and of R102A PHear (cell) to (syringe) wt μ2 linker phosphopeptide (purple).
F Coomassie staining of SDS PAGE gel of pull downs using GST and GSTPHear of Pcore. Increasing amounts of FxDxF peptide do not affect the amount of Pcore bound.
G Immunoblots of RPE cells with ectopic expression of WT NECAP1-mRuby2 (left) and R90A mutant version at near-endogenous levels. Populations of cells with similar expression levels of WT NECAP1 and R90A-NECAP1 (middle lane, right panel) were selected.
H Immunoblots of RPE cells with ectopic expression of WT or R90A NECAP1 with siRNA mediated simultaneous depletion of endogeneous NECAP1 and NECAP2 using siRNA targeting 3'UTR of NECAPs (see Fig S5C).
I Comparison of association of WT and R90A NECAP1 with endogenous AP2. Reciprocal co-immunoprecipitation of FLAG-tagged NECAP or AP2α with either FLAG epitope-specific antibody, or mouse mAb AP6 antibody. Bar graph shows quantification of the respective immunoblots (n=1).
J Time course of TfR internalization at 37°C in cells with ectopic expression of WT NECAP1-mRuby2 (left) and R90A at near-endogenous levels and simultaneous depletion of endogeneous NECAP1 and NECAP2 using siRNA targeting 3'UTR of NECAPs (see above). Data represent mean S.D., n = 3. Two-tailed Student's t-tests were used to assess statistical significance. ** $P < 0.01$.
K) Number of CCPs identified as NECAP1mRuby2-positive or NECAP1mRuby2-negative in cells, expressing of WT NECAP1-mRuby2 or R90A mutant at near-endogenous levels. Values are mean ± SD, n= 17.

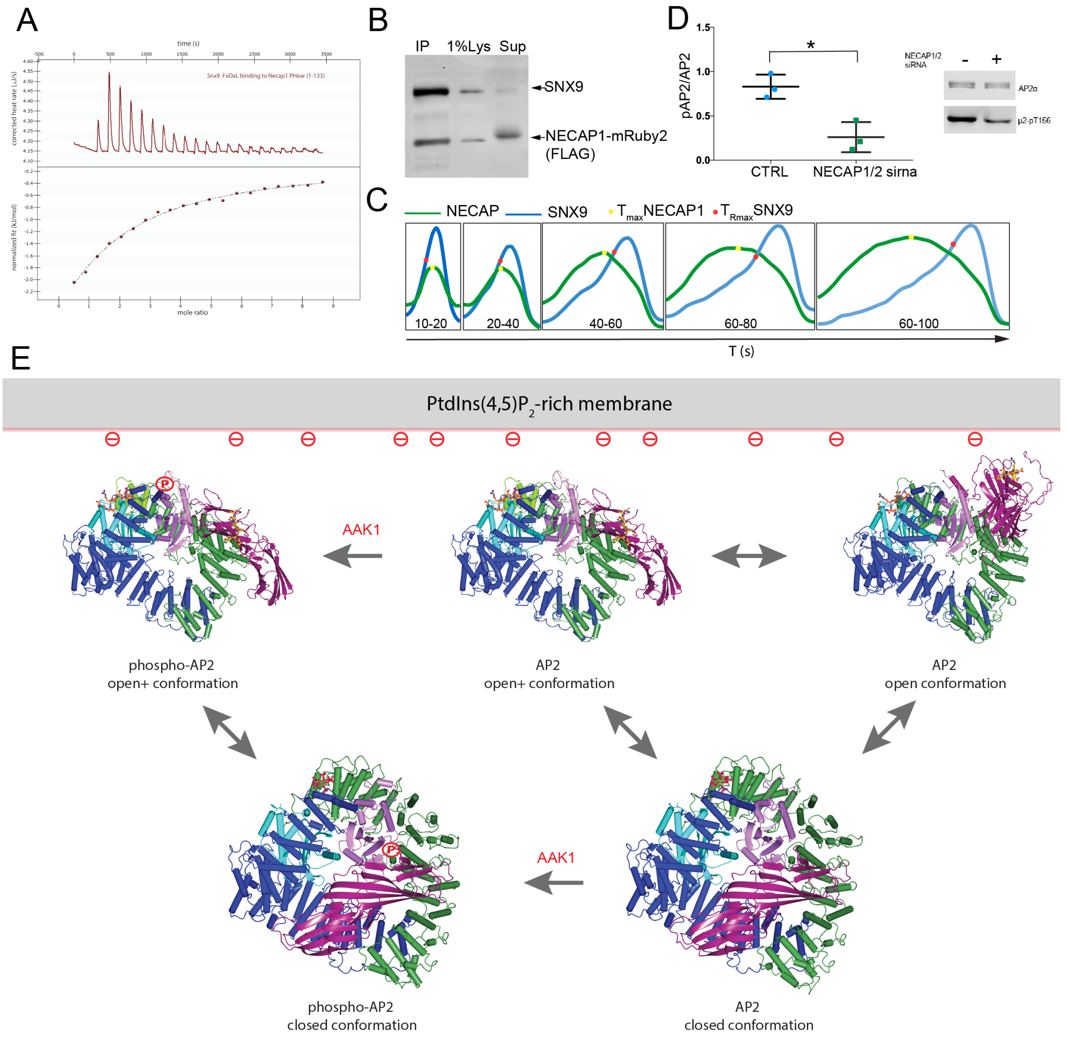

Figure S7 (related to Figure 7). P-AP2:NECAP:SNX9 interactions are important for CCP maturation.

A Example ITC trace (top) and fitted curve (bottom) of low-affinity binding of wt NECAP1 PHear (cell) to wt SNX9 linker peptide FLDSL (syringe).
B Co-immunoprecipitation of SNX9 with NECAP1-FLAG from lysate of NECAP1-mRuby2 cells prepared in 0.1% NP40.
C NECAP1 and SNX9 averaged intensity traces from 2 independent experiments were manually superimposed for different time cohorts. The red dot on the SNX9 intensity trace (blue line) represents the calculated time of maximum recruitment rate of SNX9. In comparison the time of maximum NECAP recruitment is visualized with yellow dot on NECAP intensity trace (green). The calculation of Tmax was carried out on intensity traces averaged from TIRFM acquisition of at least 17 cells expressing either NECAP1mRuby2 or SNX9mRuby2.

D Quantification of western blot band intensities of P-AP2 and AP2α. Results are represented as a relative ratio of P-AP2 and AP2 signal, n=3 .
E Schematic diagram of relationships between unphosphorylated and μ2T156 phosphorylated AP2 cores in their various conformations bound and not bound to AP2's various ligands.

Supplementary Tables

Table S1 (related to Fig. 2).

	Closed AP2 Pcore	Open+ AP2 Pcore	Open+ AP2 core
Resolution range	61-2.56 (2.62-2.56)	110-3.40 (3.5-3.40)	91-5.0 (5.59-5.0)
Rmerge	0.32 (2.564)	0.280 (6.639)	0.202 (3.60)
Rmeas	0.339 (2.708)	0.290 (6.894)	0.247 (4.42)
Rpim	0.078 (0.616)	0.077 (1.822)	0.138 (2.50)
Rmerge (top intensity bin)	0.092	0.059	0.085
Number of observations	1277705	688170	74249
Number unique	71252	49558	15525
<(I)/sd(I)>	8.4 (1.4)	8.8 (0.6)	3.6 (0.4)
CC(1/2)	0.996 (0.121)	0.993 (0.176)	0.942 (0.470)
Completeness %	99.7 (99.7)	99.9 (99.2)	99.6 (99.5)
Multiplicity	17.9 (18.8)	13.9 (13.6)	4.8 (4.6)
Wilson B ($Å^2$)	50.0	100	*Refinement*
R (Rfree)	0.226 (0.257)	0.205 (0.247)	*was*
RMS bond lengths (Å)	0.015	0.011	*not*
RMS bond angles (°)	1.8	1.7	*performed*

Crystallographic statistics for the open+ Pcore, closed Pcore, and open+ core structures.

Table S2 (related to Fig. 3).

		Core (Fast)	Core (Slow)	Pcore (Fast)	Pcore (Slow)
PIP2 only	K_D (μM)	7.4±1.2	9.5±1.3	7.3±0.9	7.8±1.2
	Percentage	49%	51%	47%	53%
	Off rate (s^{-1})	0.067±0.018	0.0065±0.0015	0.054±0.014	0.0068±0.0019
PIP2+TGN38	K_D (μM)	1.5±0.25	0.31±0.05	0.52±0.08	0.07±0.02
	Percentage	45%	55%	44%	56%
	Off rate (s^{1})	0.038±0.006	0.00037±0.00012	0.028±0.008	0.00017±0.00007
PIP2+CD4	K_D (μM)	1.5±0.3	2.6±0.5	1.9±0.3	1.8±0.2
	Percentage	65%	35%	61%	39%
	Off rate (s^{1})	0.075±0.009	0.0018±0.0003	0.058±0.012	0.0025±0.0005

Kinetic parameters of the interactions between the Pcore or core and liposomes containing PtdIns4,5P_2-only, PtdIns4,5P_2 and YxxΦ, and PtdIns4,5P_2 and[ED]xxxLL determined by liposome-based SPR.

Table S4 (related to Fig. 6) NMR structure solution statistics

**Structural restraints**	**Free Protein**	**Complex**	
NOE-derived distance restraints	**NECAP**	**NECAP**	**AP2μ**
Intraresidue	429	418	35
Sequential	947	963	46
Medium (2≤|i-j|≤4)	677	652	12
Long (|i-j|>4)	1448	1564	4
Total	3501	3597	97
Intermolecular		43	
**Statistics for accepted structures**			
Number of accepted structures	30	30	
Mean AMBER energy terms (kcal mol^{-1} ± S.D.)			
E(total)	-5530.9 ± 11.8	-6630.5 ± 11.8	
E(van der Waals)	-1051.6 ± 12.4	-1155.8 ± 9.5	
E(distance restraints)	22.8 ± 2.4	39.4 ± 2.8	
Distance restraint viols. > 0.2Å (average number per structure)	2.9 ± 1.7	8.0 ± 1.8	
RMS deviations from the ideal geometry used within AMBER			
Bond lengths	0.0098 Å	0.0101 Å	
Bond angles	1.95°	2.05°	
**Ramachandran statistics**			
	**Res. 7-131**	**Res. 7-131 & 152-162**	
Most favoured	85.0%	78.3%	
Additionally allowed	13.7%	18.9%	
Generously allowed	0.3%	2.3%	
Disallowed	1.0%	0.5%	
**Average atomic RMS deviations from the average structure (± S.D.)**			
	**Res. 7-131**	**Res. 7-131 & 152-162**	
(N, C^{α}, C' atoms)	0.61 ± 0.14 Å	0.57 ± 0.10 Å	
(All heavy atoms)	1.05 ± 0.14 Å	0.98 ± 0.09 Å	

Table S5 (related to Fig. 6).

**T156-phospho μ2 (149-163)**	NECAP1 PHear (1-133) wt	NECAP1 PHear (1-133) wt + FxDxF	NECAP1 PHear (1-133) R102A	NECAP1 PHear (1-133) R32S
n	1.01±0.06	1.07±0.11	0.96±0.03	0.93±0.03
ΔH (kJ/mol)	-24.1±1.4	-19.4±2.5	-27.4±2.4	-36.8±4.0
ΔS (J/mol*K)	-3.09±5.05	15.3±9.7	-14.8±8.3	-54.4±15.3
Kd (μM)	56.4±3.1	44.9±7.0	57.0±1.6	125±15

Thermodynamic parameters of the interactions between NECAP1 PHear domain variants and phosphorylated-μ2 linker peptides determined by ITC
